# Diagnostic Value of Serum p-tau217 in Alzheimer Disease: Equal to Plasma in Levels and Clinical Utility?

**DOI:** 10.1093/clinchem/hvaf162

**Published:** 2025-11-13

**Authors:** Andrea L Benedet, Burak Arslan, Kubra Tan, Hanna Huber, Ilaria Pola, Guglielmo Di Molfetta, Hlin Kvartsberg, Anna Orduña Dolado, Shorena Janelidze, Kaj Blennow, Henrik Zetterberg, Oskar Hansson, Pedro Rosa-Neto, Nicholas J Ashton

**Affiliations:** Department of Psychiatry and Neurochemistry, Institute of Neuroscience & Physiology, Sahlgrenska Academy at the University of Gothenburg, Mölndal, Sweden; Department of Psychiatry and Neurochemistry, Institute of Neuroscience & Physiology, Sahlgrenska Academy at the University of Gothenburg, Mölndal, Sweden; Clinical Neurochemistry Laboratory, Sahlgrenska University Hospital, Gothenburg, Sweden; Department of Psychiatry and Neurochemistry, Institute of Neuroscience & Physiology, Sahlgrenska Academy at the University of Gothenburg, Mölndal, Sweden; Department of Psychiatry and Neurochemistry, Institute of Neuroscience & Physiology, Sahlgrenska Academy at the University of Gothenburg, Mölndal, Sweden; Department of Psychiatry and Neurochemistry, Institute of Neuroscience & Physiology, Sahlgrenska Academy at the University of Gothenburg, Mölndal, Sweden; Department of Psychiatry and Neurochemistry, Institute of Neuroscience & Physiology, Sahlgrenska Academy at the University of Gothenburg, Mölndal, Sweden; Department of Psychiatry and Neurochemistry, Institute of Neuroscience & Physiology, Sahlgrenska Academy at the University of Gothenburg, Mölndal, Sweden; Clinical Memory Research Unit, Department of Clinical Sciences, Malmö, Lund University, Lund, Sweden; Clinical Memory Research Unit, Department of Clinical Sciences, Malmö, Lund University, Lund, Sweden; Department of Psychiatry and Neurochemistry, Institute of Neuroscience & Physiology, Sahlgrenska Academy at the University of Gothenburg, Mölndal, Sweden; Clinical Neurochemistry Laboratory, Sahlgrenska University Hospital, Gothenburg, Sweden; Paris Brain Institute, ICM, Pitié-Salpêtrière Hospital, Sorbonne University, Paris, France; Neurodegenerative Disorder Research Center, Division of Life Sciences and Medicine, and Department of Neurology, Institute on Aging and Brain Disorders, University of Science and Technology of China and First Affiliated Hospital of USTC, Hefei, China; Department of Psychiatry and Neurochemistry, Institute of Neuroscience & Physiology, Sahlgrenska Academy at the University of Gothenburg, Mölndal, Sweden; Clinical Neurochemistry Laboratory, Sahlgrenska University Hospital, Gothenburg, Sweden; School of Medicine and Public Health, Wisconsin Alzheimer’s Institute, University of Wisconsin, Madison, WI, United States; Department of Neurodegenerative Disease, Institute of Neurology, University College London, London, United Kingdom; UK Dementia Research Institute, University College London, London, United Kingdom; Hong Kong Center for Neurodegenerative Diseases, Hong Kong, China; Clinical Memory Research Unit, Department of Clinical Sciences, Malmö, Lund University, Lund, Sweden; Translational Neuroimaging Laboratory, Department of Neurology and Neurosurgery, Psychiatry and Pharmacology and Therapeutics, McGill University Research Centre for Studies in Aging, Montreal Neurological Institute-Hospital, Douglas Research Institute, McGill University, Montreal, Canada; Department of Psychiatry and Neurochemistry, Institute of Neuroscience & Physiology, Sahlgrenska Academy at the University of Gothenburg, Mölndal, Sweden; Banner Alzheimer’s Institute and University of Arizona, Phoenix, AZ, United States; Banner Sun Health Research Institute, Sun City, AZ, United States

## Abstract

**Background:**

Phosphorylated tau 217 (p-tau217) has emerged as a leading blood-based biomarker for Alzheimer disease (AD). While typically measured in plasma, serum is a widely used matrix in clinical laboratories, yet few p-tau217 assays have been validated for serum. Evaluating serum p-tau217 performance is essential for expanding its use in clinical and research settings, particularly for cohorts with only serum samples available.

**Methods:**

We quantified p-tau217 in plasma and serum from individuals within the AD continuum (n = 100; mean age 72.5 ± 5.0 years; 54% female) using 6 assays across 4 platforms. Spearman correlation, Passing–Bablok regression, and receiver operating characteristics analysis were used to assess intermatrix agreement and diagnostic performance. Specific validation parameters (e.g., precision, parallelism, dilution linearity, stability) were evaluated in both matrices.

**Results:**

High correlations between plasma and serum were observed for most assays (ρ > 0.8), though plasma often yielded higher concentrations. Notably, the Lumipulse assay showed near-perfect correlation (ρ = 0.98) and minimal bias. Fold changes in p-tau217 levels across the AD continuum were comparable between matrices, though cutoffs for detecting AD pathology differed. Applying plasma-derived cutoffs to serum resulted in misclassification rates ranging from 16% to 47%, except for Lumipulse (10% in serum vs 5% in plasma). Not all assays performed equally in serum, as reflected in validation metrics.

**Conclusions:**

Serum p-tau217, across multiple platforms, shows strong correlations with plasma p-tau217 and reflected comparable patterns across the AD continuum. However, absolute concentrations differed for most assays, thus requiring differing disease specific cutoffs. Most of the evaluated platforms demonstrated reliable quantification of p-tau217 in serum, yielding satisfactory validation performance. These findings support serum as a viable alternative to plasma for p-tau217 quantification in both research and clinical settings, provided matrix-specific validation is ensured.

## Introduction

In recent years, significant progress has been made in the development of diagnostic tools for Alzheimer disease (AD), enabling the detection of 2 key neuropathological hallmarks—amyloid-β (Aβ) plaques and neurofibrillary tangles—in vivo through the use of biomarkers, either with imaging [i.e., positron emission tomography (PET)] or biofluids such as cerebrospinal fluid (CSF) and more recently, blood (1). Initially, 3 PET tracers that bind to Aβ plaques (2–4) and 1 PET tracer (5) that binds to tau-containing neurofibrillary tangles have been validated against postmortem brain tissue neuropathological examinations as the reference standard and have received FDA approval (6). Following this, CSF biomarkers (i.e., Aβ_42/40_, p-tau181/Aβ_42_, and t-tau/Aβ_42_ ratios) have been validated against PET measures of amyloid, leading to their approval by both the FDA and the European Medicines Agency as diagnostic tools (7, 8). In the past few years, it has been shown that among various phosphorylated tau (p-tau) species in blood, such as p-tau181 (9), p-tau231 (10), and p-tau217 (11), consistently discriminate between Aβ-positive and Aβ-negative individuals, with higher levels observed in Aβ-positive cases, as determined using either PET or CSF Aβ markers as references, with plasma p-tau217 levels increasing in a stepwise manner through the more advanced clinical stages (11, 12). Notably, p-tau217 has been shown to outperform other p-tau species in terms of diagnostic accuracy, and p-tau217 assays correlate better among themselves (13).

The relevance of reliable blood biomarkers like p-tau217 has become even more critical with the recent FDA approval of amyloid-targeting therapies such as lecanemab (14) and donanemab (15), which are prescribed for early AD, aim to reduce brain amyloid burden, and may have their efficacy monitored through this biomarker. It is also important to emphasize that individuals with cognitive symptoms are typically first seen in primary care, and only a small percentage are referred to specialized memory clinics (1). This limited referral contributes to a significant rate of misdiagnosis, with estimates ranging from 25% to 35% in specialized clinics, and likely even higher in primary care settings, particularly among patients with symptomatic AD (1, 6). This underscores the need for improved and scalable diagnostic tools to ensure timely and accurate identification of AD in its early stages, especially given the limited access to PET and CSF biomarker testing (16).

As such, the development and implementation of accurate blood-based biomarkers—particularly those that can be integrated into routine clinical laboratory workflows—represent a promising and more accessible alternative for facilitating early diagnosis and guiding treatment decisions. Considering that routine clinical chemistry tests are already available at most primary care centers or affiliated clinical chemistry laboratories, the availability of accurate blood tests for AD becomes increasingly important for facilitating early diagnosis and potential treatment management. It is also worth mentioning that, in clinical chemistry laboratories across Europe, the choice of blood collection tubes—whether plasma or serum—for clinical chemistry tests varies between countries (17), as most tests can be reliably performed using either matrix. According to a survey conducted in 2016, plasma tubes accounted for 26% of the total clinical chemistry tubes used in Europe. Notably, countries in northern Europe, such as Finland, Sweden, and the Netherlands, reported plasma tube usage rates exceeding 50% (17).

Given the imminent clinical implementation of p-tau217 assays, it becomes crucial to define which matrix, assay, and cutoffs can be used for this biomarker. This is particularly relevant when the viability of other matrices remains uncertain, with previous studies reporting significant matrix-dependent variations in absolute biomarker concentrations (18–20), further emphasizing the need for additional research. This is especially important in light of the variability in blood tube types, as previously noted, with plasma and serum usage differing across European clinical chemistry laboratories (17). Although EDTA plasma is frequently used as the primary blood matrix in dementia biomarker research, a brief PubMed search conducted using the terms “(plasma p-tau217 OR p-tau217) AND (Alzheimer's disease OR AD)” and “(serum p-tau217) AND (Alzheimer's disease OR AD)” yielded 176 publications on plasma p-tau217 since 2020 but only 12 publications on serum p-tau217 since 2022. This disproportionate emphasis on plasma, in relation to the common use of serum in clinical practice, highlights the need for further research into the viability of serum. Previous studies have demonstrated that, although the intermatrix correlations for other p-tau species (e.g., p-tau181 and p-tau231) are strong, their concentrations can vary significantly between paired samples in different matrices (18–20), even though their diagnostic performance is generally comparable (18). Recently, Chen et al. (21) compared p-tau217 concentrations in matched EDTA plasma and serum samples across 4 assays and showed that the differences in levels varied among the assays [ALZpath, Pittsburgh, NUcleic acid Linked Immuno-Sandwich Assay (NULISA™), and Lumipulse]. However, to our knowledge, no studies have yet thoroughly investigated p-tau217 across different matrices (serum and various plasma types, including K₂EDTA, lithium heparin, and sodium citrate) using a wide range of immunoassays that may be sensitive to matrix effects. Moreover, while commercial p-tau217 assays have already undergone thorough performance evaluations by the manufacturers, additional method verification remains crucial when considering their implementation in routine clinical practice (22). This step ensures that the assays consistently perform as expected in specific laboratory settings, confirming their accuracy, precision, and reproducibility across different sample types and clinical environments.

To build on this, in this 3-phase proof-of-concept study, we first investigated p-tau217 concentrations in paired serum and K_2_EDTA plasma samples from a well-characterized AD cohort, examining intermatrix associations and comparing their diagnostic performance on 6 different p-tau217 assays [i.e., ALZpath, Janssen, Lumipulse, Meso Scale Discovery (MSD) S-PLEX, MSD-Lilly, and NULISA singleplex prototype assay]. In the second phase, experiments were conducted in plasma and serum samples to reassess selected analytical validation parameters such as lower limit of quantification (LLoQ), precision, parallelism, dilution linearity, spike recovery, and sample stability. Finally, in the third phase, blood samples were collected using 4 different collection tubes, and p-tau217 concentrations were compared across all 6 assays being evaluated.

## Materials and Methods

### Study Design

This study was designed to be performed in 3 phases. Phase I aimed at comparing plasma and serum p-tau217 concentrations from well-characterized participants ranging within the AD continuum (n = 100). Blood p-tau217 concentrations in both matrices were evaluated across pathology and diagnostic groups, and the performance of each assay in distinguishing amyloid pathology status was compared. Phase II aimed at reassessing validation parameters such as LLoQ, precision, parallelism, dilution linearity, spike recovery, and sample stability between matrices within the same assay, following standard guidelines (22–26). Finally, phase III compared blood p-tau217 quantifications on individual samples collected at the same time using blood tubes with 3 different types of additives for plasma and one for serum, leading to a final comparison between 4 different collection tubes for each p-tau217 assay evaluated (Supplemental Fig. 1).

### Ethics Approval and Consent to Participate

All participants (or legal representatives) from the TRIAD cohort provided written informed consent, and the study was approved by the Montreal Neurological Institute PET working committee and the Douglas Mental Health University Institute Research Ethics Board (MP-18-2017-157). For the anonymized samples, the collection at the Clinical Chemistry Laboratory, Sahlgrenska University Hospital was conducted in accordance with the Ethics Committee at University of Gothenburg (EPN140811).

### Study Participants

**Fig. 1.**
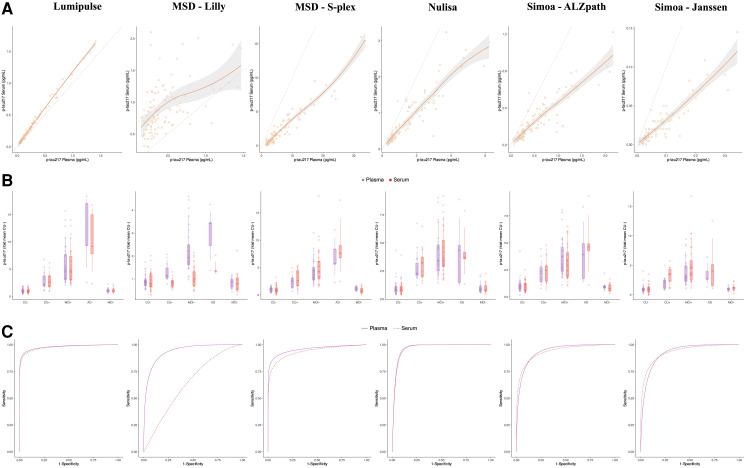
Study phase I. Phase I included 100 TRIAD participants selected by diagnosis, amyloid PET status, and availability of both plasma and serum. The target was 50% CU and 50% cognitively impaired, balanced for amyloid positivity. Final groupings were CU− (n = 35), CU+ (n = 15), MCI− (n = 15), MCI+ (n = 30), and AD dementia (n = 5). Further cohort details are in Supplemental Methods 1 and Table 1. (A), Correlation between plasma and serum p-tau217 within all the assays tested (raw values). The continuous line represents the locally estimated scatterplot smoothing (span = 1) regression and the shaded area shows the 95% CI (results detailed in Table 2). The dashed line represents the identity line; (B), Plasma and serum p-tau217 across diagnostic groups and amyloid PET status. The boxplots depict the median (horizontal bar), 25th to 75th percentiles (hinges), and whiskers indicate 10th and 90th percentiles. Plotted values represent the biomarker fold mean in relation to the CU− individuals within each blood fraction; (C), Receiver operating characteristics curves for plasma (purple, solid curve) and serum (red, dashed curve) p-tau217 differentiating among amyloid PET positive and negative individuals. Detailed reports on area under the curve and predictive values are presented in Table 3. Abbreviation: MCI, mild cognitive impairment. Color figure available at clinchem.org.

**Fig. 2.**
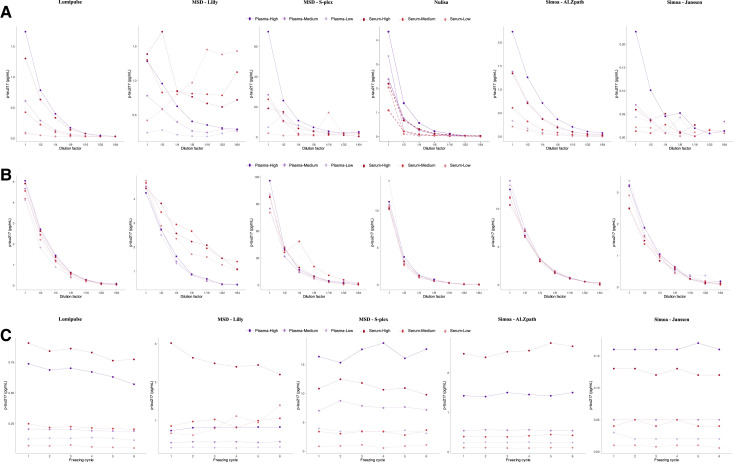
Study phase II. Phase II was conducted using anonymous samples with no clinical data for validation. (A), Concentration of p-tau217 as a function of dilution factor in the parallelism analysis. Solid purple lines represent, in different shades, low, medium, and high plasma p-tau217 levels, while red-dashed lines represent, in different shades, low, medium, and high serum p-tau217 levels; (B), Concentration of p-tau217 as a function of dilution factor in the dilution linearity analysis. Solid purple lines represent, in different shades, low, medium, and high plasma p-tau217 levels, while red-dashed lines represent, in different shades, low, medium, and high serum p-tau217 levels; (C), Concentration of p-tau217 across freeze-thaw cycles in the sample stability analysis. Solid purple lines represent, in different shades, low, medium, and high plasma p-tau217 levels, while red-dashed lines represent, in different shades, low, medium, and high serum p-tau217 levels. Color figure available at clinchem.org.

**Fig. 3.**
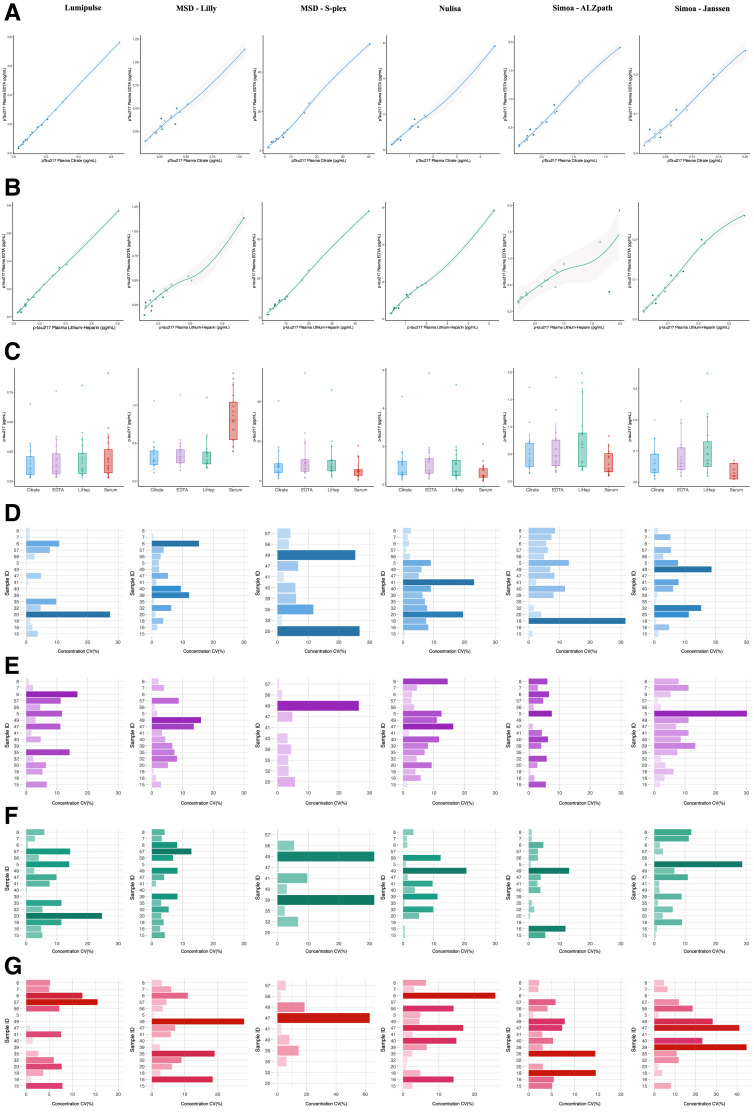
Study phase III. Phase III was conducted using anonymous samples with no clinical data for intermatrix variability assessment. (A), Correlation between p-tau217 in plasma EDTA and plasma citrate within all the assays tested (raw values). The continuous line represents the locally estimated scatterplot smoothing (span = 1) regression and the shaded area shows the 95% CI (results detailed in Supplemental Table 12); (B), Correlation between p-tau217 in plasma EDTA and plasma lithium-heparin within all the assays tested (raw values). The continuous line represents the locally estimated scatterplot smoothing (span = 1) regression and the shaded area shows the 95% CI; (C), Total plasma and serum p-tau217 across tube types within assays. The boxplots depict the median (horizontal bar), 25th to 75th percentiles (hinges), and whiskers indicate 10th and 90th percentiles (results detailed in Supplemental Table 13); (D–G), Horizontal bar plots show the CV for each assay and tube type: in blue citrate, in purple EDTA, in green lithium-heparin, and in red serum. Each bar corresponds to the CV for a given individual sample measured in duplicate (results detailed in Supplemental Table 13). Color figure available at clinchem.org.

Phase I included 100 TRIAD participants selected by diagnosis, amyloid PET status, and availability of both plasma and serum. Amyloid PET status was determined using a predefined standardized uptake value ratio threshold of 1.55, corresponding to 24 Centiloids. This quantitative classification also confirms the classification from the visual assessment from 2 blinded neurologists, ensuring consistency with clinical practice (27). The target was 50% cognitively unimpaired (CU) and 50% cognitively impaired, balanced for amyloid positivity. Final groupings were CU− (n = 35), CU + (n = 15), mild cognitive impairment negative (n = 15), mild cognitive impairment positive (n = 30), and AD dementia positive (n = 5) (Fig. 1B). Further cohort details are in Supplemental Methods 1 and Table 1. Phases II and III used anonymous samples with no clinical data for validation and intermatrix variability assessments, respectively (Figs. 2 and 3). Further information regarding cohort assessments have been previously described (27).

**Table 1.** Demographics and relevant information of the sample.

	CU− (N = 35)	CU+ (N = 15)	MCI+ (N = 30)	ADD (N = 5)	MCI− (N = 15)
Sex (female) (%)	23 (65.7)	10 (66.7)	12 (40.0)	3 (60.0)	6 (40.0)
Age, years	72.3 (4.87)	71.0 (5.71)	74.4 (4.52)	72.3 (6.62)	70.8 (4.34)
*APOE*-ε4 noncarriers (%)	28 (80.0)	7 (46.7)	9 (30.0)	1 (20.0)	8 (53.3)
*APOE*-ε4 carriers (%)	4 (11.4)	8 (53.3)	16 (53.3)	2 (40.0)	5 (33.3)
Amyloid PET, SUVR	1.27 (0.07)	1.94 (0.25)	2.42 (0.46)	2.49 (0.40)	1.28 (0.07)
Tau PET, SUVR (meta-ROI)	0.93 (0.09)	1.05 (0.18)	1.49 (0.53)	2.60 (1.04)	0.95 (0.08)

Values are given in number (percentage) or average (SD).

Abbreviations: ADD, Alzheimer’s disease dementia; MCI, mild cognitive impairment; ROI, region of interest; SUVR, standard uptake value ratio.

### Sample Collection

TRIAD samples (phase I) were collected using EDTA-treated tubes for plasma and red-top tubes for serum, processed and stored at −80°C. For phases II to III, paired serum and plasma (K_2_EDTA, lithium-heparin, sodium-citrate) were collected from anonymous participants during routine testing. All samples were aliquoted into 0.5 mL polypropylene tubes. Full procedures are detailed in Supplemental Methods 1 and Supplemental Tables 1 to 3.

### Fluid Analyses

Aliquots were prepared at the University of Gothenburg and analyzed using the following p-tau217 platforms: LUMIPULSE® G1200 (Fujirebio Europe N.V.), MSD S-PLEX, Simoa HD-X (Quanterix), and NULISA (Alamar Biosciences). Methods for ALZpath p-tau217 (11), Simoa–Janssen p-tau217 (28), MSD-Lilly p-tau217 (29), and MSD S-PLEX p-tau217 (30) have been previously described. External analyses were conducted at Lund University (MSD-Lilly) and Alamar Biosciences (NULISA). Platform-specific methods and assay characteristics are detailed in Supplemental Methods 1 and 2.

### Statistical Analyses

Statistical analyses and data plotting were performed using R statistical software (v 4.4.0). Descriptive statistics were used to present the sample set used at phase I, where c^2^ tests contrasted categorical variables and ANOVA compared continuous variables between groups. When biomarker levels were compared between groups, age and sex were included as covariates in the ANCOVA model. To facilitate comparison between fluid matrices, p-tau217 fold mean was calculated using the average biomarker levels in the CU group as reference. Spearman rank test was used to evaluate p-tau217 correlation between matrices, within assay types. The Passing–Bablok regression calculation was conducted to estimate bias and proportionality between quantifications in phase I and phase III. Biomarker accuracy in differentiating amyloid pathology status was assessed through the area under the receiver operating characteristic curve, and the 95% CI for sensitivities and specificities were calculated using the Delong test. The Youden index estimated a cutoff to differentiate these 2 groups in the whole sample set (phase I). The CV for each sample was calculated when measurements were performed in duplicate. The CV was determined using the formula (SD/mean) × 100, where the SD and mean were derived from the 2 replicate measurements. CV and sample concentrations were compared across tube types, within assays, in phase I and II using Welch 2-sample *t*-test. In phase III, similar analysis was done using the Kruskal–Wallis test, followed by post hoc analysis Dunn test with multiple testing correction using the Benjamini–Hochberg test.

## Results

### Phase I

Participant demographic characteristics are summarized in Table 1. No significant differences in age or sex were observed between groups, although cognitively impaired positive individuals had a higher prevalence of APOE ε4 carriers and more pronounced amyloid and tau pathology than CU**−** participants.

Across assays, serum p-tau217 concentrations were generally lower than those in plasma, consistent with the bias observed in the Passing–Bablok regression (Table 2, Fig. 1). Despite this, strong correlations (rho > 0.8) were observed between plasma and serum for all assays except MSD-Lilly. Lumipulse was the only platform showing comparable concentration ranges and an almost perfect correlation (rho = 0.98; CVs in Supplemental Table 4).

**Table 2.** Associations between plasma and serum quantification within assays.

Assay	Passing–Bablok regression	Intercept CI (95%)	Slope CI (95%)	Spearman rho (95% CI)
Lumipulse	S = 0.009 + 1.19*P	0.003–0.01^a^	1.15–1.24^a^	0.98 (0.96–0.99)
MSD-Lilly	S = 0.09 + 1.89*P	−0.15–0.25	1.28–2.69^a^	0.52 (0.35–0.66)
MSD S-PLEX	S = −0.54 + 0.42*P	−0.70 −0.33	0.39–0.47^a^	0.92 (0.87–0.95)
NULISA singleplex	S = 0.009 + 0.50*P	−0.03–0.03	0.46–0.56^a^	0.93 (0.88–0.96)
Simoa–ALZpath	S = −0.12 + 0.51*P	−0.02–0.007	0.44–0.56^a^	0.90 (0.83–0.93)
Simoa–Janssen	S = −0.002 + 0.36*P	−0.004–0.00	0.30–0.42^a^	0.85 (0.77–0.91)

The regression includes plasma quantifications (P) as the reference method and serum quantifications (S) as the test method. CIs at 95% were estimated using 1000 bootstrap resamples.

^a^Intercepts that are significantly different from 0 or slopes that are significantly different from 1.

Both matrices demonstrated a similar stepwise increase in p-tau217 across clinical groups (CU− to cognitively impaired positive), with similar fold mean changes within each assay (Table 3, Fig. 1). This trend was absent only for MSD-Lilly, where the stepwise increase was observed in plasma but not in serum. Serum p-tau217 also discriminated amyloid pathology with similar accuracy to plasma; however, applying plasma-derived cutoffs to serum resulted in higher misclassification rates (16%–47%) for most assays, except Lumipulse (10% in serum vs 5% in plasma; Supplemental Table 5).

**Table 3.** Assay performance across matrices in AD.

	Average fold mean (SD)				Accuracy to detect amyloid PET positivity			
Assay	CU+	MCI+	ADD	MCI-	AUC (95% CI)	PPV (95% CI)	NPV (95% CI)	Cutoff
Lumipulse plasma	2.98 (1.60)	5.73 (3.66)	11.3 (6.45)	1.09 (0.432)	0.98 (0.96-1.00)	0.97 (0.88-0.99)	0.94 (0.84-0.99)	0.14
Lumipulse serum	2.83 (1.48)	5.46 (3.30)	10.2 (5.78)	1.12 (0.430)	0.97 (0.95-1.00)	0.94 (0.83− 0.99)	0.95 (0.86–0.99)	0.17
MSD-Lilly plasma	1.43 (0.405)	2.49 (1.07)	3.31 (1.22)	0.914 (0.241)	0.94 (0.89–0.98)	0.82 (0.70–0.97)	0.95 (0.83–0.97)	0.31
MSD-Lilly serum	0.934 (0.232)	1.22 (0.503)	1.63 (0.348)	0.960 (0.523)	0.63 (0.52–0.73)	0.61 (0.46–0.75)	0.63 (0.48–0.75)	0.79
MSD S-PLEX plasma	2.43 (1.17)	4.35 (2.24)	6.77 (3.58)	1.15 (0.345)	0.96 (0.92–1.00)	1.0 (0.91–1.00)	0.82 (0.77–1.00)	5.23
MSD S-PLEX serum	2.92 (1.74)	5.32 (3.36)	8.65 (5.42)	0.846 (0.571)	0.94 (0.89–0.98)	0.97 (0.87–0.98)	0.82 (0.69–0.99)	2.04
NULISA singleplex plasma	2.81 (1.36)	3.99 (1.95)	4.14 (3.08)	0.998 (0.477)	0.968 (0.93–1.00)	0.90 (0.78–0.98)	0.95 (0.85–0.98)	1.00
NULISA singleplex serum	3.09 (1.37)	4.13 (2.10)	4.13 (2.36)	0.99 (0.48)	0.97 (0.94–1.00)	0.90 (0.78–0.98)	0.95 (0.85–0.98)	0.55
Simoa–ALZpath plasma	2.20 (1.01)	3.61 (1.79)	4.06 (2.80)	0.97 (0.37)	0.94 (0.90–0.99)	0.91 (0.80–0.96)	0.88 (0.76–0.96)	0.38
Simoa–ALZpath serum	2.41 (1.22)	3.18 (1.57)	4.84 (2.93)	0.86 (0.36)	0.93 (0.89–0.98)	0.95 (0.84–0.97)	0.84 (0.71–0.97)	0.22
Simoa–Janssen plasma	1.98 (1.07)	3.76 (2.23)	3.38 (1.91)	0.94 (0.30)	0.92 (0.87- 0.98)	0.90 (0.77–0.96)	0.84 (0.70–0.95)	0.06
Simoa–Janssen serum	3.13 (1.46)	5.05 (3.35)	4.85 (4.59)	1.20 (0.55)	0.93 (0.88–0.97)	0.89 (0.76–0.95)	0.85 (0.72–0.94)	0.01

The fold mean was calculated using the CU− individuals as reference. As such, the average of the CU− group is 1 and therefore not included in the table.

The cutoff values are intended solely for comparative purposes and should be interpreted with caution.

Abbreviations: MCI, mild cognitive impairment; ADD, AD dementia; AUC, area under the curve; PPV, positive predicted value; NPV, negative predicted value.

### Phase II

The mean p-tau217 CV% ranged from 5.2% to 12% for plasma and 5.2% to 21.3% for serum, with slightly higher variability in serum for MSD (p_Lilly_ < 0.0001; p_S-PLEX_ = 0.01) and Simoa–Janssen (*P* = 0.0003) assays (Supplemental Table 6). Most measurements were above the LLoQ in both matrices, although a few fell below for Lumipulse, NULISA, and Simoa–Janssen. All assays met acceptable performance criteria (CV% ≤ 20%) in plasma and generally in serum, with MSD S-PLEX and MSD-Lilly showing the highest variability among serum samples (Supplemental Fig. 2).

Repeatability and intermediate precision were <15% across all plasma pools except for NULISA (low) and MSD S-PLEX (intermediate). In serum, Lumipulse and Simoa–ALZpath achieved <15% variability across all levels, while Simoa–Janssen met this threshold at high concentrations (Supplemental Table 7).

Parallelism and dilution linearity were assessed up to 64x dilution. Lumipulse and Simoa–ALZpath showed consistent recoveries within the accepted 80% to 120% range across all matrices and pools (Supplemental Tables 8 and 9, Figs. 2A and B). At low levels, Lumipulse reached its LLoQ at a 4x dilution. Spike-recovery results were generally within 80% to 120%, except for NULISA (Supplemental Table 10).

Stability testing across 6 freeze–thaw cycles showed ≤20% change for most assays, confirming good sample integrity. Slight deviations were observed for MSD S-PLEX and Simoa–Janssen in low-concentration serum at cycle 4 but returned within range thereafter (Supplemental Table 11, Fig. 2C).

Detailed results are provided in Fig. 2, the Supplemental Results, and Supplemental Tables 6 to 11.

### Phase III

Plasma p-tau217 measurements were highly correlated across the different collection tubes for all assays. The least correlated measurements in this analysis stage were between plasma K_2_EDTA and plasma lithium-heparin (Fig. 3, Supplemental Table 12). Between plasma K_2_EDTA and serum, correlations followed a similar pattern to what was observed in phase I, where MSD-Lilly was the assay with the least correlation between matrices. The Passing–Bablok analysis compared p-tau217 quantifications from sodium-citrate, lithium-heparin, and serum with K_2_EDTA plasma as a reference test. In all models, the intercept was not significant, suggesting no systematic bias in relation to K_2_EDTA plasma measurements. Proportional bias was always observed between plasma K_2_EDTA and serum or plasma K_2_EDTA and plasma citrate.

No statistical differences were observed between the median measurements of the different plasma tubes (Supplemental Table 13). Serum quantifications were lower than plasma quantifications on the MSD-Lilly assay, as well as lower than plasma lithium-heparin in the Simoa assays. When comparing the p-tau217 CV% within assays, no difference was found across tube types. Samples below LLoQ, when present, were more often present when collected in sodium-citrate and serum tubes, and these agreed with each other (Supplemental Table 14).

## Discussion

This study assessed the impact of sample matrix and anticoagulant type on p-tau217 quantification across 6 assays and 4 platforms. In phase I, plasma and serum levels correlated well but differed in absolute values, indicating the need for matrix-specific cutoffs. Phase II evaluated analytical validation parameters, showing generally reliable serum performance, though some assays underperformed compared to plasma. In phase III, p-tau217 quantification in plasma samples collected using different tube types showed that anticoagulants had a negligible effect on correlations; however, differences were observed in absolute concentrations.

With the growing demand for p-tau217 assessment in research cohorts and clinical practice, factors influencing its quantification warrant thorough investigation. Most blood assays were developed for plasma, raising the question of whether serum p-tau217 can accurately determine amyloid PET positivity and whether absolute values and cutoffs are interchangeable. To explore this, we compared p-tau217 concentrations measured by leading immunoassays in matched plasma and serum samples from individuals across the AD continuum, using amyloid PET as the reference standard. As expected, plasma p-tau217 showed high accuracy, and—with the exception of MSD-Lilly—all assays performed well in serum. Strong correlations were observed between matrices, and fold mean changes from CU− individuals were similar, displaying the expected stepwise increase with clinical severity. These findings support the use of either matrix in AD research, particularly for studying core AD endophenotypes. However, plasma and serum cannot be used interchangeably, as p-tau217 concentrations were generally lower in serum and showed significant proportional bias in the Passing–Bablok analysis, indicating that plasma cutoffs cannot be directly applied to serum.

Given the initial findings, the next logical step is to examine whether the analytical validation parameters fall within acceptable limits in both plasma and serum for each assay. While these parameters have been extensively evaluated for plasma, similar assessments are still needed to confirm the reliability of quantification in serum. We acknowledge at this point that most assays included in this study have been optimized for use in plasma. Paired plasma and serum samples were tested, and all quantifications were above the reported LLoQ assigned value, with a few exceptions in the Lumipulse, NULISA, and Simoa–Jannsen results. In this sample set, %CVs were significantly higher in serum than in plasma for both the Simoa–Janssen and MSD assays, with the latter surpassing the 20% acceptable variability threshold. Similar results were observed when specifically evaluating repeatability and intermediate precision. The MSD-Lilly assay showed high variability within and between runs for all 3 levels of serum samples. At “low” p-tau217 concentrations, quantifications on serum also had high CVs when using MSD S-PLEX and the NULISA singleplex assays, which showed a tendency to improve with higher concentrations. The reduced precision and repeatability observed for the MSD assays on the 2 initial phases of this study suggests limited use of serum samples for p-tau217 quantification, as opposed to reliable quantifications in serum when using the Simoa and Lumipulse assays.

The stability of blood p-tau217 was assessed across 6 freeze–thaw cycles. Overall, p-tau217 remained stable in both plasma and serum, showing consistent reliability through at least 4 cycles across most assays. Similar stability has been reported for other p-tau isoforms. Ashton et al. (19) demonstrated that both plasma and serum p-tau181 remain stable for up to 4 freeze–thaw cycles. Similarly, studies have shown that up to 4 freeze–thaw cycles do not significantly affect p-tau217 quantifications in plasma (20, 31, 32), with one study specifically reporting that p-tau217 concentrations remained unchanged for up to 3 freeze–thaw cycles (33).

Given that laboratories and research cohorts use different sample collection protocols and blood tube types, it is important to determine whether various anticoagulant additives in plasma tubes influence biomarker quantification across platforms. The most used tube types (34) were then included in this investigation, and samples were collected at the same visit, to minimize other possible confounding factors. Overall, measurements on plasma sodium-citrate and lithium-heparin correlated extremely well with K_2_EDTA. As previously reported for other p-tau epitopes (19), citrate plasma quantifications showed a numerically lower range of p-tau217 concentrations, while those in K_2_EDTA and lithium-heparin plasma were higher. Despite these observational differences, which were also detected in the Passing–Bablok analysis, the median concentration, and %CVs, were not found to be statistically different between plasma tubes on this sample—yet, as observed with serum in phase I, different cutoffs for AD determination will apply for these blood fractions.

Although a few studies have compared the effect of different blood collection tubes on the concentration of p-tau species (19, 20, 31), the results have been inconsistent. Of these, certain studies reported that p-tau181 levels were highest in lithium heparin tubes when K_2_EDTA was used as the reference (19, 20). In contrast, one study reported that p-tau217 concentrations were lowest in lithium-heparin when K_2_EDTA was the reference (31). This discrepancy may partly be explained by the fact that handling procedures for K_2_EDTA plasma are highly standardized, whereas other tube types may require additional optimization procedures.

Another discrepancy observed in our study was the poor correlation between K_2_EDTA plasma and serum p-tau217 measurements using the MSD-Lilly assay across all 3 phases. This contrasts with recent findings from Verberk et al. (35), who reported a strong correlation between EDTA plasma and serum using the same assay. Interestingly, both studies consistently observed higher absolute p-tau217 concentrations in serum compared to plasma. These discrepancies may be explained by preanalytical factors such as differences in sample handling procedures or tube types (e.g., K_2_ vs K_3_ EDTA, serum tubes with or without gel) and warrant further investigation.

As also evidenced in a recent global plasma phospho-tau round-robin study (13), p-tau217 assays exhibit strong correlations within a given matrix, yet absolute concentrations differ significantly across platforms. Recently, Chen et al. (21) compared p-tau217 concentrations in matched serum and plasma samples using 4 assays (Lumipulse, Pittsburgh, ALZpath, and NULISA) and observed robust correlations and diagnostic performance in both matrices but notable matrix-dependent differences in absolute concentrations. Consistent with their findings, we observed strong correlations among assays in both plasma and serum yet with assay- and matrix-specific variations in absolute p-tau217 levels. Importantly, our study extends these observations by including additional platforms—MSD-Lilly, MSD S-PLEX, and Simoa–Janssen—and by performing extensive analytical validation (phase II) experiments. Moreover, in phase III, we also examined other plasma types—lithium-heparin and sodium-citrate plasma—which are commonly used in clinical laboratories and biobank collections. These results provide broader insights into preanalytical matrix effects and further support the practical implementation of p-tau217 testing across diverse clinical and research settings.

Differences in calibration materials likely contribute to discrepancies in absolute concentrations across assays and matrices. Variations in calibrator form (synthetic peptide vs recombinant protein), phosphorylation heterogeneity, and limited commutability with endogenous samples can all introduce systematic bias. Evidence from the recent plasma phospho-tau round-robin study (13) also demonstrated that several candidate reference materials were not commutable across assays, highlighting the challenge of achieving cross-platform standardization. Calibrator specifications for all assays used in this study are available in the Supplemental Material. Because calibrators lack the complex biological environment of plasma or serum, their interaction with antibodies may differ from that of native proteins, further contributing to bias. Additionally, differences in calibration curve fitting and range selection may accentuate these discrepancies. These observations reinforce the need for harmonization, and efforts by the IFCC Working Group for Biomarkers of Neurodegenerative Diseases are ongoing to standardize p-tau217 measurement through the development of reference methods and materials and subsequent commutability studies to enable cross-platform calibration and clinical translation.

Although the same procedures were rigorously followed for both plasma and serum, the protocols were optimized initially for plasma p-tau217. Therefore, future studies should examine how preanalytical variables—such as clotting time, centrifugation conditions, and the use of gel separator tubes—affect serum measurements. Such work may help optimize serum protocols and improve assay performance. These aspects are particularly relevant as blood-based p-tau217 assays move toward full automation and regulatory approval, where both plasma and serum are expected to be used.

While the findings provide important insights, our study also has limitations. First, phase I included a limited sample size (n = 100), reflecting the large sample volumes required across multiple assays. These constraints affect cutoff estimation, which should be viewed as exploratory rather than definitive. Power calculations were performed for the primary correlation analyses in phases I and III but not for the other statistical comparisons due to the exploratory nature of these analyses and limited sample availability. In addition, diagnostic comparisons with non-AD dementia cases were not included, limiting conclusions regarding assay specificity. The available sample volume also precluded duplicate testing for Lumipulse in phase I and stability assessment for NULISA; however, based on other platforms, comparable stability is expected. Finally, the phase III comparison included only 17 individuals without confirmed amyloid or cognitive status, warranting replication in larger, well-characterized cohorts. Due to logistical and commercial constraints, not all assays could be analyzed in the same laboratory: MSD-Lilly assays were performed at Lund University and NULISA singleplex at Alamar Biosciences, as previously reported.

In conclusion, this study demonstrates that p-tau217 concentrations differ between plasma and serum across assays, with plasma generally showing higher values. While most assays performed reliably in serum, reduced analytical performance in some highlights the importance of matrix-specific validation. Despite yielding comparable patterns, plasma and serum should not be used interchangeably due to absolute concentration differences—necessitating either matrix-specific cutoffs or conversion factors. Finally, p-tau217 levels showed strong agreement across plasma tube types, suggesting consistent quantification regardless of anticoagulant used. However, caution is warranted when combining data from differing collection protocols. Further studies are needed to confirm and extend these findings.

## Supplementary Material

hvaf162_Supplementary_Data

## Data Availability

The data supporting the findings of this study may be shared with qualified academic investigators for the purpose of result replication, upon reasonable request to the corresponding author and under a material transfer agreement.

## Nonstandard Abbreviations

AD, Alzheimer disease; Aβ, amyloid β; PET, positron emission tomography; CSF, cerebrospinal fluid; p-tau, phosphorylated tau; NULISA, NUcleic acid Linked Immuno-Sandwich Assay; MSD, Meso Scale Discovery; LLoQ, lower limit of quantification; CU, cognitively unimpaired.
