# Correction to: Diagnostic Value of Serum p-tau217 in Alzheimer Disease: Equal to Plasma in Levels and Clinical Utility?

**DOI:** 10.1093/clinchem/hvag015

**Published:** 2026-02-27

This is a correction to: Andrea L Benedet, Burak Arslan, Kubra Tan, Hanna Huber, Ilaria Pola, Guglielmo Di Molfetta, Hlin Kvartsberg, Anna Orduña Dolado, Shorena Janelidze, Kaj Blennow, Henrik Zetterberg, Oskar Hansson, Pedro Rosa-Neto, Nicholas J Ashton, Diagnostic Value of Serum p-tau217 in Alzheimer Disease: Equal to Plasma in Levels and Clinical Utility? *Clinical Chemistry*, 2025; https://doi.org/10.1093/clinchem/hvaf162

In the originally published version of the paper, in section **Introduction**, the last part of the first paragraph: “[…] [i.e. positron emission tomography (PET) or biofluids such as cerebrospinal fluid (CSF) and more recently blood (1–13). Initially 3 PET tracers that bind to Aβ plaques () and 1 PET tracer ().]” is corrected to read: “[…] [i.e., positron emission tomography (PET)] or biofluids such as cerebrospinal fluid (CSF) and more recently, blood (1). Initially, 3 PET tracers that bind to Aβ plaques (2–4) and 1 PET tracer (5) that binds to tau-containing neurofibrillary tangles have been validated against postmortem brain tissue neuropathological examinations as the reference standard and have received FDA approval (6). Following this, CSF biomarkers (i.e., Aβ_42_/_40_, p-tau181/Aβ_42_, and t-tau/Aβ_42_ ratios) have been validated against PET measures of amyloid, leading to their approval by both the FDA and the European Medicines Agency as diagnostic tools (7, 8). In the past few years, it has been shown that among various phosphorylated tau (p-tau) species in blood, such as p-tau181 (9), p-tau231 (10), and p-tau217 (11), consistently discriminate between Aβ-positive and Aβ-negative individuals, with higher levels observed in Aβ-positive cases, as determined using either PET or CSF Aβ markers as references, with plasma p-tau217 levels increasing in a stepwise manner through the more advanced clinical stages (11, 12). Notably, p-tau217 has been shown to outperform other p-tau species in terms of diagnostic accuracy, and p-tau217 assays correlate better among themselves (13).”

The article has been corrected.

