# Supplementary material for: Diagnostic Value of Serum p-tau217 in Alzheimer Disease: Equal to Plasma in Levels and Clinical Utility?
Source: Clin Chem. 2025 Nov 13;72(2):303–15. doi: 10.1093/clinchem/hvaf162 (PMC12865796; doi:10.1093/clinchem/hvaf162)
Supplement: hvaf162_Supplementary_Data [file hvaf162_Supplementary_Data.docx]

**Diagnostic Value of Serum p-tau217 in Alzheimer Disease: Equal to Plasma in Levels and Clinical Utility?**

**Running header title: Plasma vs. Serum p-tau217 in AD Diagnostics**

Andrea L. Benedet*^1^, Burak Arslan*^1^, Kubra Tan^1^, Hanna Huber^1^, Ilaria Pola^1^, Guglielmo Di Molfetta^1^, Hlin Kvartsberg^1^, Anna Orduña Dolado^9^, Shorena Janelidze^9^, Kaj Blennow^1,2,3,4^, Henrik Zetterberg^1,2,5,6,7,8^, Oskar Hansson^9^, Pedro Rosa-Neto^10^, Nicholas J. Ashton^1,11,12^

**Affiliations:**

^1^ Department of Psychiatry and Neurochemistry, Institute of Neuroscience & Physiology, the Sahlgrenska Academy at the University of Gothenburg, Mölndal, Sweden

^2^Clinical Neurochemistry Laboratory, Sahlgrenska University Hospital, Gothenburg, Sweden

^3^ Paris Brain Institute, ICM, Pitié-Salpêtrière Hospital, Sorbonne University, Paris, France

^4^ Neurodegenerative Disorder Research Center, Division of Life Sciences and Medicine, and Department of Neurology, Institute on Aging and Brain Disorders, University of Science and Technology of China and First Affiliated Hospital of USTC, Hefei, P.R. China

^5^ Wisconsin Alzheimer’s Institute, School of Medicine and Public Health, University of Wisconsin, Madison, WI, USA

^6^ Department of Neurodegenerative Disease, Institute of Neurology, University College London, London, UK

^7^ UK Dementia Research Institute, University College London, London, UK

^8^ Hong Kong Center for Neurodegenerative Diseases, Hong Kong, China

^9^Clinical Memory Research Unit, Department of Clinical Sciences, Malmö, Lund University, Lund, Sweden

^10^Translational Neuroimaging Laboratory, Department of Neurology and Neurosurgery, Psychiatry and Pharmacology and Therapeutics, McGill University Research Centre for Studies in Aging, Montreal Neurological Institute-Hospital, Douglas Research Institute, McGill University, Montreal, Canada

^11^ Banner Alzheimer’s Institute and University of Arizona, Phoenix, Arizona, USA

^12^ Banner Sun Health Research Institute, Sun City, Arizona, USA

* These authors contributed equally to this work

**SUPPLEMENTARY METHODS**

**Supplementary methods 1.** Sample Collection, study participants, and fluid analyses

For *Phase I*, TRIAD whole blood samples were collected according to clinical best practices, using commercial ethylenediaminetetraacetic acid (EDTA)-treated tubes for plasma and red-topped tubes for serum (during the same visit). After collection, the red-topped tubes were left at room temperature for approximately 30 minutes to clot undisturbed, while the EDTA tubes were gently inverted several times to ensure proper mixing of blood with the anticoagulant. Both types of tubes were centrifuged at 2200g for 10 minutes at 20°C. The resulting plasma and serum were transferred into 1mL polypropylene tubes and stored at -80°C until analysis. Samples were transported on dry ice to the Clinical Neurochemistry Laboratory at Sahlgrenska University Hospital in Mölndal, Sweden, for further analysis.

In *Phase II*, paired serum samples (n = 75; collected using BD Vacutainer SST™ II Advance tubes, REF 366566, LOT 4100730) and K_2_EDTA plasma samples (collected using VACUETTE® K2EDTA tubes, REF 456243, LOT A24023Q7) were obtained at the routine blood-drawing site of the Clinical Chemistry Laboratory at Sahlgrenska University Hospital from participants undergoing routine blood test assessments. After collection, the serum separator tubes were left undisturbed at room temperature for approximately 30 minutes to allow clotting, while the EDTA tubes were gently inverted several times to ensure proper mixing of the blood with the anticoagulant. Both types of tubes were centrifuged at 2200g for 10 minutes at 20°C. The resulting plasma and serum were aliquoted into 0.5 mL polypropylene tubes, with volumes appropriate for use in each platform and analysis, and stored at -80°C until analysis.

For *Phase III*, paired serum (n = 17; collected using BD Vacutainer SST™ II Advance tubes, REF 366566, LOT 4100730), K2EDTA plasma (collected using VACUETTE® K2EDTA tubes, REF 456243, LOT A24023Q7), lithium-heparin (collected using VACUETTE® Lithium heparin tubes, REF 456305, LOT A2409458), and sodium-citrate (collected using BD Vacutainer buffered sodium citrate REF 364305 LOT 4205650)) plasma samples were obtained at the routine blood-drawing site of the Clinical Chemistry Laboratory at Sahlgrenska University Hospital from participants undergoing routine blood test assessments. After collection, the serum separator tubes were left undisturbed at room temperature for approximately 30 minutes to allow clotting, while the EDTA, lithium-heparin, and sodium-citrate tubes were gently inverted several times to ensure proper mixing of the blood with their respective anticoagulants. All tubes were centrifuged at 2200g for 10 minutes at 20°C. The resulting plasma and serum were aliquoted into 0.5 mL polypropylene tubes, with volumes appropriate for use in each platform, and stored at -80°C until analysis.

On the day of analysis, in all study phases, the samples were thawed at room temperature (RT), vortexed at 2000 rpm for 30 seconds, and then centrifuged at 4000g for 10 minutes at 20°C.

The *Phase I* consisted of samples from 100 individuals from the TRIAD cohort, which were selected based on their clinical diagnosis, amyloid pathology status (defined by PET imaging using the [18F]NAV4694 tracer with a cutoff for positivity of 1.55 SUVR, which is equivalent to 24 Centiloids) and availability of plasma and serum samples. It was intended that from the 100 individuals, 50% were cognitively unimpaired (CU) and 50% with cognitive impairment (CI). Similarly, it was intended that 50% of the set were positive and 50% negative for amyloid pathology. Thus, the final population of analyses for this study phase comprised of 35 cognitively unimpaired individuals amyloid negative (CU-), 15 cognitively unimpaired amyloid positive individuals (CU+), 15 mild cognitively impaired amyloid negative individuals (MCI-), 30 mild cognitively impaired amyloid positive individuals (MCI+) and 5 amyloid positive individuals with the clinical diagnosis of AD dementia (ADD). A detailed description of the selected individuals is presented in Table 1. Further information regarding cohort assessments have been previously described (Reference 26 in the main manuscript).

*Phase* *II* consisted of randomly collected paired plasma and serum samples from anonymous participants with no available clinical data. These samples were used to reassess the selected validation parameters. Detailed methods regarding Phase II are provided in the Supplementary Methods.

Similarly to *Phase II*, *Phase III* consisted of randomly collected paired samples from 17 anonymous participants with no available clinical data. These samples were used to assess the inter-matrix variability of p-tau217.

Sample aliquoting was performed simultaneously for all assays at the Department of Neurochemistry, University of Gothenburg. Two sets of aliquots, designated for MSD Lilly p-tau217 and NULISA p-tau217 measurements across all phases, were sent to external centers: the Clinical Memory Research Unit at Lund University and Alamar Biosciences (Freemont, U.S), respectively. The remaining aliquots were analyzed at the Department of Neurochemistry, University of Gothenburg. All samples were analyzed for p-tau217 using the following platforms: LUMIPULSE^®^ G1200 (Fujirebio Europe N.V., Ghent, Belgium), Meso Scale Discovery (MSD) S-PLEX (Rockville, MD, USA), Simoa HD-X (Quanterix, Billerica, MA, USA) and NUcleic acid Linked Immuno-Sandwich Assay (NULISA™, Alamar Biosciences, Fremont, CA, USA). Methods for ALZpath p-tau217 (Reference 11 in the main manuscript), Janssen Simoa p-tau217 (Reference 27 in the main manuscript), MSD Lilly p-tau217 (Reference 28 in the main manuscript), and MSD S-PLEX p-tau217 (Reference 29 in the main manuscript), have been previously described. Detailed descriptions of the methods for Fujirebio Lumipulse G p-tau217 and NULISA Singleplex p-tau217 prototype assay are included in the Supplementary Methods 2. Comprehensive descriptions of the pre-analytical and analytical phases for each study phase are provided below.

In *Phase I*, individual matched plasma and serum samples were analyzed side by side in the same run, in duplicate. In *Phase II*, the evaluation focused on selected validation parameters, which included the LLoQ, precision, parallelism, dilution linearity, spike recovery, and sample stability. Sample collection and preparation were carried out prior to the assessment of these parameters, with the rationale for their inclusion detailed in the Supplementary Methods 3. For *Phase III*, all individual matched plasma and serum samples were analyzed side by side in the same run, with duplicate quantifications conducted to evaluate coefficient of variation (CV) and examine inter-matrix variability. Detailed procedures are provided in Supplementary Methods 4.

**Supplementary methods 2.** Assay description and specifications.

**Alamar Biosciences NULISA Singleplex p-tau217 prototype assay**

Alamar Biosciences NULISA Singleplex p-tau217 prototype assay was performed at Alamar Biosciences, as previously described (1). Briefly, plasma and serum samples stored at -80°C were thawed on ice and centrifuged at 10,000g for 10 minutes. The singleplex p-tau217 NULISA qPCR assay utilized 20 µL of sample per reaction with duplicate measurements. A Hamilton-based automation instrument was used to execute the NULISA workflow, beginning with immunocomplex formation using DNA-barcoded capture and detection antibodies. Subsequent steps involved capturing and washing the immunocomplexes on paramagnetic oligo-dT beads, releasing them into a low-salt buffer, and further capturing and washing on streptavidin beads. The proximal ends of DNA strands on each immunocomplex were ligated using T4 DNA ligase, generating a DNA reporter. In the single-plex qPCR assays, molecule quantification was performed through qPCR reactions (2). For the phase 2 experiments that required sample dilution(e.g., parallelism, dilution linearity), the NULISA Sample Dilution Buffer (PN: 801041, LN: 2402204) was used.

**Fujirebio Lumipulse G p-tau217**

Plasma and serum p-tau217 levels were measured using a Lumipulse G pTau 217 Plasma RUO assay on the LUMIPULSE G1200 system at the Department of Neurochemistry, University of Gothenburg. Briefly, plasma and serum samples stored at -80°C were thawed at room temperature. After vortexing at 2000 rpm for 20 seconds, the samples were centrifuged at 2000 g for 10 minutes. This assay employs a two-step process, where the analyte is initially captured in the presence of an assay-specific solution on RD85-coated particles. Following a washing step, detection is achieved using an ALP-labeled HT7/BT2 conjugate. A synthetic peptide containing the three target epitopes serves as the calibrator. Plasma and serum samples were analyzed in singlicate, aliquoted a total sample volume of 225 µL, including the dead volume of the sample cup. For duplicate measurements, a total sample volume of 325 µL was aliquoted, accounting for the dead volume and the additional measurement(2). The required volume used by the instrument is stated in Table 1.

**Assay specifications**

All samples from phases I, II, and III were analyzed using appropriate reagents and assay kits. The following assays were utilized: ALZpath (ALZpath Simoa p-tau217 v2 assay kit, REF: 104371, LOT: 999048), Janssen (Simoa ACC p-tau217 assay kit, REF: 104730, LOT: 999049), MSD S-PLEX (S-PLEX Human Tau (p-tau217) kit, catalog number: K151APFS-series, LOT: K00S0173), and Lumipulse G. For the Lumipulse G assay, cartridge LOT D4C4061 and substrate LOT 4081 were used in phases I and III, while cartridge LOT D4C5022 and substrate LOT 5016 were used in phase II.

**Calibrator specifications**

The calibrators used in this study differed across assays in terms of molecular composition and production source. The calibrator sources for each assay are as follows:

Lumipulse: synthetic p-tau217 peptide; MSD Lilly: synthetic p-tau217 peptide; MSD S-PLEX: recombinant p-tau217 expressed in a human cell line and verified by mass spectrometry to confirm phosphorylation at T217; Nulisa Singleplex: full-length recombinant tau-441 with site-specific phosphorylation at T217; Simoa ALZpath: synthetic peptide; Simoa Janssen: synthetic peptide (4.5 kDa) corresponding to the capture antibody epitope (AB-PEG4-epitope of detection antibody).

**Supplementary methods 2.** *Phase II.*

In this phase, partial validation was conducted to assess the analytical performance of several p-tau217 assays for both serum and plasma matrices. Since all the assays were intended for research use only and were not developed in-house, the validation followed a partial approach, as previously described by Andreasson et al. (3). Furthermore, the standard ISO 15189 (4), designed for clinical laboratories, provides limited guidance, merely stating that 'the validations shall be as extensive as necessary to meet the needs in the given application or field of application.' Consequently, the choice of validation parameters was guided by the aim to include as many relevant parameters as possible, while being constrained by the limited sample volumes obtained from anonymous participants. The selected validation parameters included the Lower Limit of Quantification (LLoQ), precision, parallelism, dilution linearity, spike recovery, and sample stability. Sample collection and preparation were performed prior to evaluating the selected validation parameters, and the rationale for their inclusion and the reasons for their selection are briefly explained below.

**Sample collection and preparation**

In Phase 2, paired serum samples (n = 75; collected using BD Vacutainer SST™ II Advance tubes, REF 366566, LOT 4100730) and K_2_EDTA plasma samples (collected using VACUETTE® K2EDTA tubes, REF 456243, LOT A24023Q7) were obtained at the routine blood-drawing site of the Clinical Chemistry Laboratory at Sahlgrenska University Hospital from the same participants undergoing routine blood test assessments. After collection, the yellow-topped serum separator tubes were left undisturbed at room temperature for approximately 30 minutes to allow clotting, while the EDTA tubes were gently inverted several times to ensure proper mixing of the blood with the anticoagulant. Both types of tubes were centrifuged at 2200g for 10 minutes at 20°C. The resulting plasma and serum were aliquoted into 0.5 mL polypropylene tubes, with volumes appropriate for each platform and verification parameter.

After aliquoting 75 plasma and serum samples for LLoQ verification into polypropylene (PP) tubes, an additional set of aliquots was prepared to estimate the approximate concentration of each sample. These concentrations were used to create three levels of pools for both plasma and serum: low, medium, and high. Based on the measured concentrations, plasma and serum samples were combined in 50 mL Sarstedt screw-cap tubes (polypropylene, conical base, sterile). The initial concentrations of the individual samples were measured using the ALZpath immunoassay on the Simoa platform, and the resulting pools were designated for precision, parallelism, dilution linearity, and spike recovery experiments (3). The results of these pools are presented in Table 2.

Since the initial sample pool was insufficient for stability testing, an additional set of 75 non-paired plasma and serum leftover samples was collected from the Clinical Chemistry Laboratory at Sahlgrenska University Hospital. After quantifying the concentrations of these samples, three new pools were created for both plasma and serum, as described previously. The quantification of these pools was performed using the Lumipulse G1200 platform with p-tau217 immunocartridges. These pools were designated for the sample stability experiments, and the results are presented in Table 3.

**Selected validation parameters**

**Lower limit of quantification (LLoQ)**

To determine the lowest concentration of the analyte measurable with acceptable levels of precision and accuracy, 75 randomly collected paired plasma and serum samples (without any associated clinical information) were analyzed in duplicate for each assay included in this study. The rationale for using randomly collected samples was to simulate a real-world scenario where p-tau217 is implemented as a biomarker in routine clinical laboratories, reflecting conditions under which laboratories receive and process samples without prior knowledge of clinical context. Since no samples exceeded the upper limit of quantification and the aim was to assess native samples (without any spiking), it was not possible to evaluate the upper limit of quantification under this parameter. To assess this parameter, the samples were run in duplicates, and the average concentration and %CV (coefficient of variation) were calculated for each sample. Scatter plots were then created to visualize %CV as a function of concentration for all samples. These scatter plots were visually evaluated to identify any samples with high %CV. The LLoQ was determined by identifying the highest mean concentration level at which the %CV was <20% for most samples and verified. The summary of LLoQ verification is presented in Table 4.

**Repeatability and intermediate precision**

These parameters were tested using three different levels (low, medium, and high) of plasma pools and three levels (low, medium, and high) of serum pools, as described previously. After creating the pools for plasma and serum, 25 aliquots of each pool were prepared and stored at -80°C until analysis. On five different days, five replicates of each pool were measured using each assay. Repeatability and intermediate precision were then calculated using a macro previously developed and defined, based on the formulas outlined in ISO 5725-2(5) . The precision values for each assay are summarized in Table 5.

**Parallelism**

For this experiment, no spiking was performed; instead, samples with endogenous concentrations of the analyte were used from three different levels (low, medium, and high) of plasma and serum pools. These samples were diluted with the dilution buffer specific to each assay. Neat and serially diluted samples were analyzed in duplicate within the same run, with results adjusted for the dilution factor. For each sample, the %CV was calculated based on the results from the neat sample and its corresponding dilutions. The results for each assay are shown in Table 6.

**Dilution Linearity**

This parameter was assessed by spiking three different plasma and serum pools with cerebrospinal fluid (CSF) at the highest possible concentrations. To ensure consistency, the same spiking material (CSF pool) was used throughout the experiment. A CSF pool was collected, and its concentration was determined prior to the spiking experiment. The concentration of the CSF pool was measured at 20.9 pg/mL using the ALZpath immunoassay on the Simoa platform before the experiment. These samples were then diluted with the dilution buffer specific to each assay. Serial dilutions were prepared in vials for both parallelism and dilution linearity experiments and analyzed in duplicate, with results adjusted for the dilution factor. %Recovery was calculated for the measured concentration at each dilution. The results for each assay are shown in Table 7.

**Spike recovery**

For this experiment, plasma and serum pools at three distinct concentrations were used. All samples were spiked with the same spiking material (CSF pool) was used for this experiment. The concentration of the CSF pool was measured at 20.9 pg/mL using the ALZpath immunoassay on the Simoa platform before the experiment. Both neat and spiked samples were analyzed in the same run. For the calculation of recovery following formula is used. The results for each assay are shown in Table 8.

% Recovery = $Measured concentration\div Expected concentration \times100$

**Sample stability**

For the freeze-thaw stability experiment, plasma and serum pools at three different concentration levels were used. Each pool was divided into six aliquots of equal volume. The aliquots (#1–6) were initially stored at −80°C. Subsequently, the following steps were performed:

- Aliquot #1 was retained at −80°C without additional processing.
- Aliquots #2–6 were thawed for 2 hours at room temperature and then returned to −80°C for at least 12 hours.
- Aliquots #3–6 were subjected to a second freeze-thaw cycle.
- Aliquots #4–6 underwent a third freeze-thaw cycle.
- Aliquots #5–6 were subjected to a fourth freeze-thaw cycle.
- Aliquot #6 was subjected to a fifth freeze-thaw cycle.
- Finally, all aliquots for a given sample were thawed simultaneously and analyzed in duplicate within the same assay run.

**Supplementary methods 3.** *Phase III*.

In Phase III, paired serum (n = 17; collected using BD Vacutainer SST™ II Advance tubes, REF 366566, LOT 4100730), K2EDTA plasma (collected using VACUETTE® K2EDTA tubes, REF 456243, LOT A24023Q7), lithium-heparin plasma, and sodium-citrate plasma samples were obtained at the routine blood-drawing site of the Clinical Chemistry Laboratory at Sahlgrenska University Hospital from the same participants undergoing routine blood test assessments. After collection, the yellow-topped serum separator tubes were left undisturbed at room temperature for approximately 30 minutes to allow clotting, while the EDTA, lithium-heparin, and sodium-citrate tubes were gently inverted several times to ensure proper mixing of the blood with their respective anticoagulants. All tubes were centrifuged at 2200g for 10 minutes at 20°C. The resulting plasma and serum were aliquoted into 0.5 mL polypropylene tubes, with volumes appropriate for each platform.

**Supplementary results**

*Phase II*

Across all assays, the mean p-tau217 CV% ranged from 5.18% to 12% for plasma and from 5.22% to 21.30% for serum. The CV% was higher in serum, as compared to plasma, for the MSD assays (p_Lilly_ < 0.0001, p_S-PLEX_ = 0.01) as well as for the Simoa–Janssen assay (p = 0.0003; Supplementary Table 6). Most measurements were above the reported LLoQ for each assay in both plasma and serum; however, some samples fell below the LLoQ for Lumipulse, NULISA, and Simoa–Janssen assays (Supplementary Table 6). Overall, all assays evaluated in this LLoQ assessment demonstrated acceptable performance in plasma, as indicated by the CV% derived from duplicate measurements (arbitrarily defined as 20%), and similarly in serum, although with a few important considerations. MSD S-PLEX p-tau217 showed relatively high but still acceptable CV% for plasma (mean CV% = 12.0) and a higher CV% for serum (20.1%). Additionally, MSD Lilly demonstrated low CV% for plasma (mean CV% = 5.18) but a higher mean CV% for serum (21.30%). Janssen p-tau217 also exhibited a high but still acceptable CV% for serum (mean CV% = 16.74%) (Supplementary Table 6, Supplementary Figure 1).

When evaluating precision parameters, repeatability (%CV_r_) and intermediate precision (%CV_Rw_) were assessed. Across three plasma pool levels of known levels of “low”, “intermediate” and “high” levels of p-tau217, all assays demonstrated %CV_r_ and %CV_Rw_ below 15%, except for the NULISA Singleplex in the low concentration pool and the MSD S-PLEX assay in the intermediate pool. In contrast, for serum pools at the same concentration levels, Lumipulse and Simoa-ALZpath demonstrated %CV_r_ and %CV_Rw_ below 15% across all three pools. Additionally, in the high concentration pool, the Simoa-Janssen assay also met this criterion. Full precision data are presented in Supplementary Table 7.

In the parallelism and dilution linearity experiments, serial dilutions were performed up to 64x. Overall, across all assays, the parallelism experiments showed that Lumipulse and ALZPath demonstrated acceptable performance (with recoveries within the conventionally accepted range of 80–120%) for both sample types and all three pool levels (Supplementary Table 8 and Figure 2a). However, at low concentrations, Lumipulse reached its LLoQ after a dilution factor of 4, where further dilution did not yield a quantifiable result for both plasma and serum. A summary of the parallelism experiments is provided in Supplementary Table 8. Consistent with the parallelism experiment, dilution linearity experiments showed Lumipulse and ALZPath demonstrated acceptable performance across both sample types and all three pool levels (Supplementary Table 9 and Figure 2b). Spike recovery was also assessed, and overall, with a few exceptions, all assays—except NULISA Singleplex—demonstrated acceptable performance within the conventionally accepted range of 80–120%. Detailed results of this experiment can be found in Supplementary Table 10.

In general, p-tau217 for each assay demonstrated good stability up to six sample freeze-thaw cycles, remaining within the acceptable limit of a 20% change (calculated as the difference from the average across cycles; Supplementary Table 11 and Figure 2c). However, a few exceptions were observed—most notably MSD S-PLEX and Simoa-Janssen in low-concentration serum samples at the fourth cycle—where recovery exceeded the 20% limit, despite falling within acceptable limits (<20%) at the fifth and sixth cycles. Absolute concentrations for each freeze-thaw cycle and the percentage difference from the average concentration are presented in Supplementary Table 11 and Figure 2c.

Results for *Phase II* are presented in Figure 2, the Supplementary Results, Supplementary Tables 6-11, and Supplementary Figure 1.

**SUPPLEMENTARY TABLES**

**Supplementary Table 1.** Platform-Specific Aliquoted and Required Volumes for Validation

| **Platform** | **Aliquoted volume (µL)** | **Aspirated volume (µL)** |
| --- | --- | --- |
| Lumipulse | 325 | 100 |
| MSD - Lilly | 120 | 60 |
| MSD - S-PLEX | 70 | 50 |
| NULISA Singleplex | 80 | 50 |
| Simoa - ALZpath | 100 | 80 |
| Simoa - Janssen | 240 | 220 |

For all the selected validation parameters, the final volume was targeted to match the aliquoted volume specified above in µL. All aliquoting was performed using 0.5 mL SARSTEDT screw-cap, skirted, sterile, conical-base polypropylene (PP) tubes. Additionally, the required volume for each immunoassay, as shown in the table, accounts for duplicate measurements.

**Supplementary Table 2.** Concentrations of Plasma and Serum Pools for Validation Experiments

| **Pool** | **Concentration (pg/mL)** |
| --- | --- |
| Plasma Low | 0.35 |
| Plasma Medium | 1.37 |
| Plasma High | 2.44 |
| Serum Low | 0.22 |
| Serum Medium | 0.61 |
| Serum High | 1.47 |

The concentrations of plasma and serum pools designated as low, medium, and high were prepared by combining individual samples based on their initial concentrations measured using the ALZpath immunoassay on the Simoa platform. These pools were used for precision, parallelism, dilution linearity, and spike recovery experiments. The low p-tau217 levels for the low pools were targeted and defined based on a binary reference point for amyloid-beta (Aβ) positivity using the Youden index (0.42 pg/mL), as previously published by Ashton et al (6). This approach ensures that the data can be generalized to real-world scenarios

**Supplementary Table 3.** Concentrations of Plasma and Serum Pools for Sample Stability Experiments

| **Pool** | **Concentration (pg/mL)** |
| --- | --- |
| Plasma Low | 0.156 |
| Plasma Medium | 0.255 |
| Plasma High | 0.829 |
| Serum Low | 0.103 |
| Serum Medium | 0.265 |
| Serum High | 1.5 |

The concentrations of plasma and serum pools designated as low, medium, and high were prepared by combining individual samples based on their initial concentrations measured using the Lumipulse p-tau217 immunoassay on the Lumipulse G1200 platform. These pools were used only for sample stability experiments. The low p-tau217 levels for the low pools were targeted and defined based on a binary reference point for amyloid-beta (Aβ) positivity using the Youden index (0.212 pg/mL).

**Supplementary Table 4.** Assays CV and LLOQ values on study *Phase I*.

| **Assay** | **Plasma CV** | **Serum CV** | **LLOQ value** | **Number of plasma samples below LLOQ** | **Number of**  **serum samples**  **below LLOQ** |
| --- | --- | --- | --- | --- | --- |
| Lumipulse^*^ | NA | NA | 0.03 | 1 | 1 |
| MSD - Lilly | 5.37 (5.52) | 8.22 (9.05) | 0.18 | 2 | 0 |
| MSD – S-PLEX | 9.42 (8.58) | 19.9 (22.8) | 0.006 | 0 | 0 |
| NULISA Singleplex | 6.23 (5.29) | 6.30 (4.76) | 0.295 | 3 | 24 |
| Simoa - ALZpath | 9.35 (10.7) | 6.88 (7.94) | 0.007 | 0 | 0 |
| Simoa - Janssen | 9.71 (15.0) | 16.1 (15.1) | 0.0038 | 0 | 8 |

Unless otherwise specified, mean (SD) values are given. If the mean sample concentration was below the LLOQ, the CV was not computed in this calculation.

^*^Due to limited sample volume available, duplicate measurements could not be performed for the Lumipulse assay at this stage.

*Abbreviations: CV, coefficient of variation; LLOQ, lower limit of quantification; NA, not available.*

**Supplementary Table 5.** Cutoff application in study *Phase I*.

| **Assay** | **Matrix** | **Cutoff*** | **False Positive** | **False negative** | **Misclassified** | **AUC (CI)** |
| --- | --- | --- | --- | --- | --- | --- |
| Lumipulse^*^ | Plasma | 0.14 | 3 | 2 | 5% | 0.95 (0.90-0.99) |
|  | Serum |  | 8 | 2 | 10% | 0.90 (0.84-0.95) |
| MSD - Lilly | Plasma | 0.31 | 12 | 1 | 13% | 0.87 (0.80-0.93) |
|  | Serum |  | 47 | 0 | 47% | 0.53 (0.49-0.56) |
| MSD – S-PLEX | Plasma | 5.23 | 0 | 6 | 6% | 0.94 (0.89-0.98) |
|  | Serum |  | 0 | 35 | 35% | 0.65 (0.58-0.71) |
| NULISA  Singleplex | Plasma | 1.00 | 2 | 5 | 7% | 0.92 (0.87-0.98) |
|  | Serum |  | 1 | 15 | 16% | 0.83 (0.76-0.90) |
| Simoa - ALZpath | Plasma | 0.38 | 6 | 5 | 11% | 0.89 (0.82-0.95) |
|  | Serum |  | 0 | 27 | 27% | 0.73 (0.66-0.79) |
| Simoa - Janssen | Plasma | 0.06 | 4 | 8 | 12% | 0.87 (0.80-0.93) |
|  | Serum |  | 0 | 41 | 41% | 0.58 (0.52-0.63) |

* The cutoff was estimated for plasma and applied in serum.

*Abbreviations: AUC, area under the receiver operating characteristic curve; CI, confidence interval; CV, coefficient of variation.*

**Supplementary Table 6.** The summary of the LLoQ verification experiments.

|  | **PLASMA (n =75)** | | | | **SERUM (n= 75)** | | | | |
| --- | --- | --- | --- | --- | --- | --- | --- | --- | --- |
| **Assay** | **LLoQ (pg/mL)** | **Below**  **LLoQ (n)** | **Mean c. (pg/mL)** | **Mean CV %** | | **LLoQ (pg/mL)** | **Below**  **LLoQ (n)** | **Mean c. (pg/mL)** | **Mean CV %** |
| Lumipulse | 0.03 | 4 | 0.117 | 8.44 | | 0.03 | 3 | 0.133 | 6.60 |
| MSD Lilly | 0.18 | 1 | 0.338 | 5.18 | | 0.18 | 0 | 1.157 | 21.30* |
| MSD S-PLEX | 0.006 | 0 | 5.904 | 12.0 | | 0.006 | 0 | 3.721 | 20.10* |
| NULISA Singleplex | 0.295 | 6 | 5.794 | 9.21 | | 0.295 | 26 | 7.878 | 11.26 |
| Simoa -ALZpath | 0.007 | 0 | 0.326 | 5.38 | | 0.007 | 0 | 0.227 | 5.22 |
| Simoa - Janssen | 0.0038 | 0 | 0.042 | 8.14 | | 0.0038 | 5 | 0.018 | 16.74* |

Paired (n = 75) plasma and serum samples were analyzed in duplicate to calculate the %CV for each assay. The table presents the functional LLoQ values stated by different manufacturers. The 'Below LLoQ' column indicates the number of samples that fell below the stated LLoQ. 'Mean c.' and 'Mean %CV' represent the average concentrations and %CV of the 75 samples, respectively.

*Indicates the assays in which CVs are statistically higher in serum as compared to plasma.

*Abbreviations: CV, coefficient of variation.*

**Supplementary Table 7.** Summary of precision experiments.

|  | **PLASMA** | | | | | | | | | |
| --- | --- | --- | --- | --- | --- | --- | --- | --- | --- | --- |
|  | **Low** | | | **Intermediate** | | | **High** | | |  |
| **Assay** | **Mean** | **%CV_r_** | **%CV_Rw_** | **Mean** | **%CV_r_** | **%CV_Rw_** | **Mean** | **%CV_r_** | **%CV_Rw_** |  |
| Lumipulse | 0.1 | 6.5 | 6.9 | 0.8 | 3.0 | 3.8 | 1.8 | 2.2 | 3.4 |  |
| MSD – Lilly | 0.3 | 9.6 | 13.2 | 0.8 | 2.4 | 4.1 | 1.5 | 5.3 | 5.5 |  |
| MSD –S-PLEX | 6.8 | 9.0 | 12.7 | 27.1 | 15.7 | 16.2 | 58.7 | 10.6 | 13.0 |  |
| NULISA Singleplex | 5.1 | 30.3 | 47.5 | 3.8 | 7.5 | 12.6 | 6.4 | 9.0 | 15.5 |  |
| Simoa – ALZpath | 0.3 | 5.2 | 5.2 | 1.4 | 5.5 | 7.9 | 2.5 | 4.1 | 5.5 |  |
| Simoa – Janssen | 0.04 | 9.8 | 14.1 | 0.1 | 6.3 | 9.7 | 0.4 | 5.3 | 9.3 |  |
|  | **SERUM** | | | | | | | | | |
|  |  | **Low** |  | **Intermediate** | | | **High** | | |  |
| **Assay** | **Mean** | **%CV_r_** | **%CV_Rw_** | **Mean** | **%CV_r_** | **%CV_Rw_** | **Mean** | **%CV_r_** | **%CV_Rw_** |  |
| Lumipulse | 0.1 | 7.1 | 11.5 | 0.5 | 3.0 | 4.1 | 1.4 | 2.6 | 3.0 |  |
| MSD – Lilly | 0.9 | 26.8 | 47.5 | 1.3 | 32.6 | 34.9 | 1.1 | 21.0 | 21.9 |  |
| MSD – S-PLEX | 2.5 | 42.6 | 43.5 | 6.1 | 12.3 | 22.6 | 19.7 | 8.3 | 18.6 |  |
| NULISA Singleplex | 2.6 | 10.1 | 46.0 | 1.6 | 9.7 | 22.2 | 3.3 | 6.3 | 19.4 |  |
| Simoa – ALZpath | 0.2 | 6.5 | 8.2 | 0.6 | 6.9 | 10.3 | 1.4 | 7.1 | 9.3 |  |
| Simoa – Janssen | 0.01 | 18.0 | 21.3 | 0.05 | 9.7 | 16.3 | 0.1 | 5.9 | 11.9 |  |

Three levels of plasma and serum samples were used to perform precision experiments, as described in the Precision section of this supplementary material. Mean” indicates the average concentration of the sample pool for both plasma and serum at each of the three levels, expressed in pg/mL. '%CV_r_' represents repeatability, while '%CV_Rw_' represents intermediate precision.

*Abbreviations: CV, coefficient of variation.*

**Supplementary Table 8.** The Summary of the Parallelism Experiments

| **Dilution rate** | **Sample Type** | | **Mean Count/Signal/AEB** | **CV%** | **Result** | **Corrected result** | **Remarks** | **Recovery%** |
| --- | --- | --- | --- | --- | --- | --- | --- | --- |
| **Lumipulse** |  | |  |  |  |  |  |  |
| 1 | Plasma Low | | 1714 | 7.07 | 0.100 | 0.100 |  |  |
| 2 | Plasma Low | | 1373 | 5.89 | 0.048 | 0.096 |  | 96.00 |
| 4 | Plasma Low | | 1265 | 25.93 | 0.030 | 0.120 | GL | N/A |
| 8 | Plasma Low | | 1167.5 | N/A | 0.030 | 0.240 | GL | N/A |
| 16 | Plasma Low | | 1115 | N/A | 0.030 | 0.480 | GL | N/A |
| 32 | Plasma Low | | 1124 | N/A | 0.030 | 0.960 | GL | N/A |
| 64 | Plasma Low | | 1162.5 | N/A | 0.030 | 1.920 | GL | N/A |
| 1 | Plasma Med | | 3639 | 40.09 | 0.609 | 0.609 |  |  |
| 2 | Plasma Med | | 2461 | 29.75 | 0.290 | 0.580 |  | 95.32 |
| 4 | Plasma Med | | 1689 | 31.55 | 0.130 | 0.520 |  | 85.46 |
| 8 | Plasma Med | | 1402 | 27.44 | 0.067 | 0.536 |  | 88.09 |
| 16 | Plasma Med | | 1176 | 29.77 | 0.038 | 0.608 | GL | N/A |
| 32 | Plasma Med | | 1164 | N/A | 0.030 | 0.960 | GL | N/A |
| 64 | Plasma Med | | 1124 | N/A | 0.030 | 1.920 | GL | N/A |
| 1 | Plasma High | | 10482.5 | 0.12 | 1.740 | 1.740 |  |  |
| 2 | Plasma High | | 5479 | 2.25 | 0.785 | 1.569 |  | 90.20 |
| 4 | Plasma High | | 3442 | 7.42 | 0.400 | 1.600 |  | 91.98 |
| 8 | Plasma High | | 2118 | 3.29 | 0.172 | 1.376 |  | 79.10 |
| 16 | Plasma High | | 1546.5 | 1.81 | 0.078 | 1.248 |  | 71.74 |
| 32 | Plasma High | | 1358 | 9.03 | 0.047 | 1.504 |  | 86.46 |
| 64 | Plasma High | | 1252 | 8.84 | 0.032 | 2.048 | GL | N/A |
| 1 | Serum Low | | 1550.5 | 13.51 | 0.079 | 0.080 |  |  |
| 2 | Serum Low | | 1362 | 8.84 | 0.048 | 0.100 |  | 122.29 |
| 4 | Serum Low | | 1182.5 | N/A | 0.030 | 0.120 | GL | N/A |
| 8 | Serum Low | | 1170.5 | N/A | 0.030 | 0.240 | GL | N/A |
| 16 | Serum Low | | 1119 | N/A | 0.030 | 0.480 | GL | N/A |
| 32 | Serum Low | | 1116 | N/A | 0.030 | 0.960 | GL | N/A |
| 64 | Serum Low | | 1111.5 | N/A | 0.030 | 1.920 | GL | N/A |
| 1 | Serum Med | | 3639 | 3.66 | 0.425 | 0.425 |  |  |
| 2 | Serum Med | | 2461 | 3.48 | 0.224 | 0.447 |  | 105.18 |
| 4 | Serum Med | | 1689 | 1.39 | 0.102 | 0.408 |  | 96.00 |
| 8 | Serum Med | | 1402 | 12.86 | 0.050 | 0.396 |  | 93.18 |
| 16 | Serum Med | | 1176 | N/A | 0.030 | 0.480 | GL | N/A |
| 32 | Serum Med | | 1164 | N/A | 0.030 | 0.960 | GL | N/A |
| 64 | Serum Med | | 1124 | N/A | 0.030 | 1.920 | GL | N/A |
| 1 | Serum High | | 8155.5 | 2.23 | 1.303 | 1.303 |  |  |
| 2 | Serum High | | 4689.5 | 5.15 | 0.632 | 1.264 |  | 97.04 |
| 4 | Serum High | | 3074.5 | 4.44 | 0.335 | 1.338 |  | 102.73 |
| 8 | Serum High | | 1919.5 | 1.02 | 0.139 | 1.112 |  | 85.37 |
| 16 | Serum High | | 1539.5 | 0.00 | 0.077 | 1.232 |  | 94.59 |
| 32 | Serum High | | 1170 | N/A | 0.030 | 0.960 | GL | N/A |
| 64 | Serum High | | 1151.5 | N/A | 0.030 | 1.920 | GL | N/A |
| **MSD – Lilly** |  | |  |  |  |  |  |  |
| 1 | Plasma Low | | 131 | 2.09 | 0.243 | 0.243 |  |  |
| 2 | Plasma Low | | 142 | 0.90 | 0.281 | 0.562 |  | 231.04 |
| 4 | Plasma Low | | 123 | 3.59 | 0.213 | 0.850 |  | 349.69 |
| 8 | Plasma Low | | 117 | 1.33 | 0.191 | 1.528 |  | 628.28 |
| 16 | Plasma Low | | 116 | 4.08 | 0.187 | 2.998 |  | 1232.85 |
| 32 | Plasma Low | | 128 | 6.57 | 0.232 | 7.436 |  | 3057.97 |
| 64 | Plasma Low | | 139 | 3.74 | 0.272 | 17.404 |  | 7156.83 |
| 1 | Plasma Med | | 281 | 0.65 | 0.780 | 0.780 |  |  |
| 2 | Plasma Med | | 227 | 4.30 | 0.587 | 1.174 |  | 150.59 |
| 4 | Plasma Med | | 174 | 2.55 | 0.397 | 1.590 |  | 203.94 |
| 8 | Plasma Med | | 138 | 2.86 | 0.267 | 2.132 |  | 273.52 |
| 16 | Plasma Med | | 132 | 3.11 | 0.245 | 3.920 |  | 502.77 |
| 32 | Plasma Med | | 145 | 6.09 | 0.292 | 9.334 |  | 1197.28 |
| 64 | Plasma Med | | 138 | 13.26 | 0.268 | 17.173 |  | 2202.81 |
| 1 | Plasma High | | 422 | 3.13 | 1.281 | 1.740 |  |  |
| 2 | Plasma High | | 331 | 2.89 | 0.956 | 1.569 |  | 90.20 |
| 4 | Plasma High | | 238 | 2.42 | 0.626 | 1.600 |  | 91.98 |
| 8 | Plasma High | | 177 | 3.12 | 0.406 | 1.376 |  | 79.10 |
| 16 | Plasma High | | 162 | 2.86 | 0.354 | 1.248 |  | 71.74 |
| 32 | Plasma High | | 150 | 5.74 | 0.310 | 1.504 |  | 86.46 |
| 64 | Plasma High | | 145 | 0.87 | 0.292 | 2.048 |  | 117.73 |
| 1 | Serum Low | | 184 | 1.80 | 0.420 | 0.421 |  |  |
| 2 | Serum Low | | 227 | 1.74 | 0.580 | 1.152 |  | 273.66 |
| 4 | Serum Low | | 292 | 30.08 | 0.800 | 3.215 |  | 763.65 |
| 8 | Serum Low | | 339 | 45.03 | 0.970 | 7.761 |  | 1843.24 |
| 16 | Serum Low | | 476 | 12.55 | 1.450 | 23.191 |  | 5508.03 |
| 32 | Serum Low | | 455 | 21.46 | 1.380 | 44.036 |  | 10459.12 |
| 64 | Serum Low | | 470 | 4.13 | 1.430 | 91.435 |  | 21716.77 |
| 1 | Serum Med | | 433 | 2.65 | 1.300 | 1.300 |  |  |
| 2 | Serum Med | | 298 | 5.42 | 0.827 | 1.654 |  | 127.26 |
| 4 | Serum Med | | 303 | 1.48 | 0.843 | 3.372 |  | 259.40 |
| 8 | Serum Med | | 294 | 2.15 | 0.811 | 6.490 |  | 499.30 |
| 16 | Serum Med | | 291 | 2.80 | 0.801 | 12.811 |  | 985.58 |
| 32 | Serum Med | | 286 | 0.00 | 0.785 | 25.115 |  | 1932.12 |
| 64 | Serum Med | | 382 | 14.99 | 1.122 | 71.789 |  | 5522.78 |
| 1 | Serum High | | 458 | 12.61 | 1.385 | 1.385 |  |  |
| 2 | Serum High | | 552 | 3.58 | 1.711 | 3.423 |  | 247.13 |
| 4 | Serum High | | 304 | 6.45 | 0.848 | 3.393 |  | 244.97 |
| 8 | Serum High | | 280 | 2.61 | 0.764 | 6.109 |  | 441.09 |
| 16 | Serum High | | 253 | 2.63 | 0.667 | 10.664 |  | 769.93 |
| 32 | Serum High | | 239 | 2.85 | 0.617 | 19.741 |  | 1425.26 |
| 64 | Serum High | | 268 | 28.84 | 0.719 | 46.028 |  | 3323.18 |
| **MSD – S-PLEX** | |  |  |  |  |  |  |  |
| 1 | Plasma Low | | 326 | 1.73 | 6.557 | 6.557 |  |  |
| 2 | Plasma Low | | 657 | 87.12 | 15.408 | 30.817 |  | 469.97 |
| 4 | Plasma Low | | 172 | 21.62 | 2.444 | 9.778 |  | 149.11 |
| 8 | Plasma Low | | 136 | 20.34 | 1.484 | 11.870 |  | 181.02 |
| 16 | Plasma Low | | 130 | 4.32 | 1.310 | 20.965 |  | 319.73 |
| 32 | Plasma Low | | 132 | 62.24 | 1.364 | 43.642 |  | 665.56 |
| 64 | Plasma Low | | 159 | 98.66 | 2.085 | 133.417 |  | 2034.67 |
| 1 | Plasma Med | | 1130 | 6.41 | 28.044 | 28.044 |  |  |
| 2 | Plasma Med | | 467 | 4.76 | 10.325 | 20.651 |  | 73.64 |
| 4 | Plasma Med | | 371 | 21.22 | 7.746 | 30.985 |  | 110.49 |
| 8 | Plasma Med | | 237 | 4.52 | 4.180 | 33.439 |  | 119.23 |
| 16 | Plasma Med | | 192 | 50.70 | 2.978 | 47.655 |  | 169.93 |
| 32 | Plasma Med | | 185 | 59.50 | 2.792 | 89.333 |  | 318.54 |
| 64 | Plasma Med | | 133 | NaN | 3.032 | 194.026 |  | 691.86 |
| 1 | Plasma High | | 2690 | 0.65 | 69.821 | 69.821 |  |  |
| 2 | Plasma High | | 986 | 1.95 | 24.192 | 48.383 |  | 69.295 |
| 4 | Plasma High | | 489 | 1.21 | 10.900 | 43.600 |  | 62.445 |
| 8 | Plasma High | | 323 | 3.22 | 6.464 | 51.709 |  | 74.060 |
| 16 | Plasma High | | 224 | 1.97 | 3.833 | 61.323 |  | 87.828 |
| 32 | Plasma High | | 176 | 8.18 | 2.538 | 81.210 |  | 116.311 |
| 64 | Plasma High | | 209 | 23.10 | 3.432 | 219.660 |  | 314.602 |
| 1 | Serum Low | | 159 | 15.27 | 2.578 | 2.578 |  |  |
| 2 | Serum Low | | 108 | 13.60 | 0.986 | 1.973 |  | 76.52 |
| 4 | Serum Low | | 118 | 78.16 | 1.281 | 5.125 |  | 198.77 |
| 8 | Serum Low | | 110 | 6.48 | 1.034 | 8.271 |  | 320.79 |
| 16 | Serum Low | | 618 | 118.40 | 16.224 | 259.585 |  | 10068.03 |
| 32 | Serum Low | | 121 | 76.65 | 1.391 | 44.509 |  | 1726.28 |
| 64 | Serum Low | | 90 | 28.82 | 0.396 | 25.352 |  | 983.27 |
| 1 | Serum Med | | 911 | 8.22 | 25.016 | 25.016 |  |  |
| 2 | Serum Med | | 435 | 100.20 | 10.858 | 21.717 |  | 86.81 |
| 4 | Serum Med | | 144 | 2.08 | 2.114 | 8.455 |  | 33.80 |
| 8 | Serum Med | | 118 | 6.83 | 1.302 | 10.413 |  | 41.62 |
| 16 | Serum Med | | 120 | 28.00 | 1.348 | 21.570 |  | 86.23 |
| 32 | Serum Med | | 163 | 100.92 | 2.670 | 85.428 |  | 341.49 |
| 64 | Serum Med | | 150 | 82.07 | 2.275 | 145.604 |  | 582.04 |
| 1 | Serum High | | 707 | 5.17 | 19.010 | 19.010 |  |  |
| 2 | Serum High | | 628 | 32.83 | 16.662 | 33.324 |  | 175.30 |
| 4 | Serum High | | 261 | 3.01 | 5.701 | 22.805 |  | 119.96 |
| 8 | Serum High | | 197 | 16.20 | 3.748 | 29.985 |  | 157.73 |
| 16 | Serum High | | 147 | 57.25 | 2.187 | 34.993 |  | 184.08 |
| 32 | Serum High | | 118 | 20.49 | 1.301 | 41.643 |  | 219.06 |
| 64 | Serum High | | 94 | 127.30 | 0.519 | 33.227 |  | 174.79 |
| NULISA Singleplex |  | |  |  |  |  |  |  |
| 1 | Plasma Low | | 1091.08 | 23.04 | 3.336 | 3.336 |  |  |
| 2 | Plasma Low | | 78.45 | 52.77 | 0.218 | 0.436 |  | 13.06 |
| 4 | Plasma Low | | 25.04 | 65.22 | 0.048 | 0.193 |  | 5.79 |
| 8 | Plasma Low | | 21.68 | 89.82 | 0.037 | 0.296 |  | 8.87 |
| 16 | Plasma Low | | 18.64 | 3.89 | 0.027 | 0.436 |  | 13.07 |
| 32 | Plasma Low | | 15.18 | 71.80 | 0.016 | 0.520 |  | 15.59 |
| 64 | Plasma Low | | 16.25 | 61.65 | 0.020 | 1.248 |  | 37.42 |
| 1 | Plasma Med | | 777.10 | 11.11 | 2.377 | 2.377 |  |  |
| 2 | Plasma Med | | 248.35 | 9.77 | 0.749 | 1.499 |  | 63.04 |
| 4 | Plasma Med | | 89.29 | 17.96 | 0.252 | 1.008 |  | 42.40 |
| 8 | Plasma Med | | 47.89 | 15.19 | 0.121 | 0.968 |  | 40.72 |
| 16 | Plasma Med | | 25.37 | 26.56 | 0.049 | 0.788 |  | 33.15 |
| 32 | Plasma Med | | 20.17 | 45.69 | 0.033 | 1.04 |  | 43.75 |
| 64 | Plasma Med | | 17.61 | 8.84 | 0.024 | 1.536 |  | 64.61 |
| 1 | Plasma High | | 1424.28 | 0.59 | 4.350 | 4.350 |  |  |
| 2 | Plasma High | | 453.54 | 4.11 | 1.384 | 2.768 |  | 63.63 |
| 4 | Plasma High | | 188.74 | 9.16 | 0.564 | 2.254 |  | 51.82 |
| 8 | Plasma High | | 79.72 | 16.24 | 0.222 | 1.776 |  | 40.83 |
| 16 | Plasma High | | 46.97 | 37.37 | 0.118 | 1.892 |  | 43.50 |
| 32 | Plasma High | | 21.22 | 12.86 | 0.036 | 1.144 |  | 26.30 |
| 64 | Plasma High | | 10.66 | N/A | 0.005 | 0.288 |  | 6.62 |
| 1 | Serum Low | | 666.30 | 0.78 | 2.038 | 2.038 |  |  |
| 2 | Serum Low | | 43.13 | 6.67 | 0.106 | 0.212 |  | 10.40 |
| 4 | Serum Low | | 22.07 | 5.51 | 0.039 | 0.154 |  | 7.56 |
| 8 | Serum Low | | 21.47 | 14.43 | 0.037 | 0.294 |  | 14.43 |
| 16 | Serum Low | | 14.19 | N/A | 0.030 | 0.472 |  | 23.16 |
| 32 | Serum Low | | 13.12 | 89.32 | 0.010 | 0.304 |  | 14.92 |
| 64 | Serum Low | | 10.12 | N/A | 0.004 | 0.224 |  | 10.99 |
| 1 | Serum Med | | 358.24 | 2.95 | 1.090 | 1.090 |  |  |
| 2 | Serum Med | | 78.68 | 23.76 | 0.219 | 0.438 |  | 40.15 |
| 4 | Serum Med | | 40.30 | 5.83 | 0.097 | 0.388 |  | 35.60 |
| 8 | Serum Med | | 25.03 | 0.73 | 0.048 | 0.386 |  | 35.42 |
| 16 | Serum Med | | 17.23 | 33.82 | 0.023 | 0.368 |  | 33.77 |
| 32 | Serum Med | | 11.39 | N/A | N/A | N/A |  | N/A |
| 64 | Serum Med | | 9.63 | 10.88 | 0.003 | 0.208 |  | 19.09 |
| 1 | Serum High | | 664.57 | 2.68 | 2.194 | 2.194 |  |  |
| 2 | Serum High | | 200.53 | 2.00 | 0.671 | 1.341 |  | 61.12 |
| 4 | Serum High | | 94.95 | 6.95 | 0.310 | 1.241 |  | 56.56 |
| 8 | Serum High | | 40.52 | 3.29 | 0.118 | 0.946 |  | 43.12 |
| 16 | Serum High | | 29.57 | 16.21 | 0.079 | 1.256 |  | 57.25 |
| 32 | Serum High | | 17.80 | 2.02 | 0.035 | 1.120 |  | 51.05 |
| 64 | Serum High | | 16.00 | 13.77 | 0.028 | 1.808 |  | 82.41 |
| **Simoa – ALZpath** | | |  |  |  |  |  |  |
| 1 | Plasma Low | | 0.10668457 | 0.09 | 0.334 | 0.334 |  |  |
| 2 | Plasma Low | | 0.05816106 | 0.60 | 0.174 | 0.348 |  | 104.42 |
| 4 | Plasma Low | | 0.03270426 | N/A | 0.089 | 0.356 |  | 106.80 |
| 8 | Plasma Low | | 0.01795393 | 2.98 | 0.039 | 0.310 |  | 93.03 |
| 16 | Plasma Low | | 0.01367654 | 3.44 | 0.024 | 0.383 |  | 114.84 |
| 32 | Plasma Low | | 0.01006354 | 26.74 | 0.011 | 0.358 |  | 107.41 |
| 64 | Plasma Low | | 0.00854484 | 46.78 | 0.006 | 0.367 |  | 110.03 |
| 1 | Plasma Med | | 0.43549551 | 9.74 | 1.381 | 1.381 |  |  |
| 2 | Plasma Med | | 0.2330524 | 4.00 | 0.741 | 1.481 |  | 107.27 |
| 4 | Plasma Med | | 0.12104141 | 12.06 | 0.380 | 1.521 |  | 110.14 |
| 8 | Plasma Med | | 0.06986733 | 5.89 | 0.213 | 1.703 |  | 123.32 |
| 16 | Plasma Med | | 0.03445727 | 3.57 | 0.095 | 1.519 |  | 110.04 |
| 32 | Plasma Med | | 0.02397715 | 26.78 | 0.059 | 1.901 |  | 137.70 |
| 64 | Plasma Med | | 0.01829398 | N/A | 0.040 | 2.557 |  | 185.22 |
| 1 | Plasma High | | 0.70721968 | 5.02 | 2.228 | 2.228 |  |  |
| 2 | Plasma High | | 0.39666565 | N/A | 1.259 | 2.518 |  | 112.99 |
| 4 | Plasma High | | 0.22432193 | 1.05 | 0.713 | 2.851 |  | 127.94 |
| 8 | Plasma High | | 0.11691724 | 1.26 | 0.367 | 2.935 |  | 131.71 |
| 16 | Plasma High | | 0.06782264 | 9.82 | 0.206 | 3.298 |  | 147.99 |
| 32 | Plasma High | | 0.03828112 | 3.57 | 0.108 | 3.451 |  | 154.85 |
| 64 | Plasma High | | 0.02867733 | 5.16 | 0.075 | 4.828 |  | 216.65 |
| 1 | Serum Low | | 0.06963956 | 3.59 | 0.212 | 0.212 |  |  |
| 2 | Serum Low | | 0.04350345 | 2.04 | 0.125 | 0.251 |  | 118.18 |
| 4 | Serum Low | | 0.02405411 | 9.25 | 0.060 | 0.239 |  | 112.60 |
| 8 | Serum Low | | 0.01530327 | 30.93 | 0.030 | 0.237 |  | 111.61 |
| 16 | Serum Low | | 0.0104809 | 26.01 | 0.013 | 0.203 |  | 95.66 |
| 32 | Serum Low | | 0.00788627 | 106.02 | 0.003 | 0.106 |  | 49.92 |
| 64 | Serum Low | | 0.00692395 | N/A | 0.002 | 0.102 |  | 48.31 |
| 1 | Serum.Med | | 0.19259238 | 4.03 | 0.611 | 0.611 |  |  |
| 2 | Serum.Med | | 0.10301499 | 16.97 | 0.322 | 0.643 |  | 105.22 |
| 4 | Serum.Med | | 0.04960194 | 16.57 | 0.146 | 0.583 |  | 95.35 |
| 8 | Serum Med | | 0.03103592 | 22.74 | 0.083 | 0.667 |  | 109.16 |
| 16 | Serum Med | | 0.01958034 | 22.71 | 0.044 | 0.710 |  | 116.18 |
| 32 | Serum Med | | 0.01286172 | 37.31 | 0.021 | 0.674 |  | 110.32 |
| 64 | Serum Med | | 0.01035521 | 14.55 | 0.012 | 0.783 |  | 128.17 |
| 1 | Serum High | | 0.42315528 | 13.80 | 1.342 | 1.342 |  |  |
| 2 | Serum High | | 0.22261886 | 4.37 | 0.707 | 1.414 |  | 105.40 |
| 4 | Serum High | | 0.11806132 | 2.42 | 0.371 | 1.482 |  | 110.45 |
| 8 | Serum High | | 0.06284416 | 4.48 | 0.190 | 1.517 |  | 113.06 |
| 16 | Serum High | | 0.03683579 | 17.90 | 0.103 | 1.647 |  | 122.75 |
| 32 | Serum High | | 0.02154492 | 7.68 | 0.051 | 1.636 |  | 121.92 |
| 64 | Serum High | | 0.01550106 | 23.96 | 0.030 | 1.938 |  | 144.43 |
| **Simoa – Janssen** | | |  |  |  |  |  |  |
| 1 | Plasma Low | | 0.00227985 | N/A | 0.044 | 0.044 |  |  |
| 2 | Plasma Low | | 0.00197098 | 50.37 | 0.033 | 0.067 |  | 153.66 |
| 4 | Plasma Low | | 0.00073541 | N/A | 0.010 | 0.041 | <Blank | 93.33 |
| 8 | Plasma Low | | 0.00152595 | N/A | 0.043 | 0.342 | <Blank | 784.91 |
| 16 | Plasma Low | | 0.00093909 | 135.91 | 0.003 | 0.049 | <Blank | 113.42 |
| 32 | Plasma Low | | 0.00094389 | N/A | 0.017 | 0.539 | <Blank | 1236.94 |
| 64 | Plasma Low | | 0.00130369 | 14.94 | 0.012 | 0.795 |  | 1825.61 |
| 1 | Plasma Med | | 0.00301585 | 10.59 | 0.070 | 0.070 |  |  |
| 2 | Plasma Med | | 0.00214081 | 25.68 | 0.039 | 0.078 |  | 111.52 |
| 4 | Plasma Med | | 0.00119217 | 91.01 | 0.010 | 0.038 | <Blank | 55.02 |
| 8 | Plasma Med | | 0.00130585 | 45.25 | 0.013 | 0.101 |  | 144.11 |
| 16 | Plasma Med | | 0.00071398 | N/A | 0.002 | 0.031 | <Blank | 45.08 |
| 32 | Plasma Med | | 0.00034135 | N/A | N/A | N/A | <Blank | N/A |
| 64 | Plasma Med | | 0.0012989 | N/A | 0.034 | 2.173 | <Blank | 3113.69 |
| 1 | Plasma High | | 0.00687907 | 19.48 | 0.227 | 0.227 |  |  |
| 2 | Plasma High | | 0.00384236 | 25.45 | 0.101 | 0.202 |  | 89.21 |
| 4 | Plasma High | | 0.00232855 | 17.80 | 0.045 | 0.181 |  | 79.81 |
| 8 | Plasma High | | 0.00253007 | 11.87 | 0.052 | 0.418 |  | 184.32 |
| 16 | Plasma High | | 0.0015325 | 78.79 | 0.020 | 0.315 |  | 138.63 |
| 32 | Plasma High | | 0.00085525 | N/A | 0.008 | 0.271 | <Blank | 119.40 |
| 64 | Plasma_High | | 0.00132923 | 110.49 | 0.014 | 0.887 | <Blank | 390.97 |
| 1 | Serum Low | | 0.00155937 | 128.49 | 0.022 | 0.022 | <Blank |  |
| 2 | Serum Low | | 0.00097624 | N/A | 0.021 | 0.041 | <Blank | N/A |
| 4 | Serum Low | | 0.00115002 | 95.71 | 0.008 | 0.034 | <Blank | N/A |
| 8 | Serum Low | | 0.00092989 | 99.54 | 0.003 | 0.022 | <Blank | N/A |
| 16 | Serum Low | | 0.00124542 | 59.34 | 0.011 | 0.174 | <Blank | N/A |
| 32 | Serum Low | | 0.0010381 | N/A | 0.017 | 0.540 | <Blank | N/A |
| 64 | Serum Low | | 0.00095096 | N/A | 0.009 | 0.556 | <Blank | N/A |
| 1 | Serum Med | | 0.00133617 | 29,87 | 0.013 | 0.013 |  |  |
| 2 | Serum Med | | 0.00118029 | 82.49 | 0.009 | 0.018 | <Blank | N/A |
| 4 | Serum Med | | 0.00100362 | N/A | 0.027 | 0.109 | <Blank | N/A |
| 8 | Serum Med | | 0.00121583 | 139.99 | 0.011 | 0.089 | <Blank | N/A |
| 16 | Serum Med | | 0.00074477 | N/A | 0.003 | 0.042 | <Blank | N/A |
| 32 | Serum Med | | 0.00105532 | N/A | 0.015 | 0.484 | <Blank | N/A |
| 64 | Serum Med | | 0.00072725 | N/A | N/A | N/A | <Blank | N/A |
| 1 | Serum High | | 0.00271107 | 61.53 | 0.060 | 0.060 |  |  |
| 2 | Serum High | | 0.00207069 | 36.47 | 0.037 | 0.073 |  | 123.08 |
| 4 | Serum High | | 0.00247987 | 41.51 | 0.051 | 0.203 |  | 341.40 |
| 8 | Serum High | | 0.00119071 | N/A | 0.009 | 0.074 |  | 124.19 |
| 16 | Serum High | | 0.00176857 | 36.39 | 0.027 | 0.427 |  | 717.53 |
| 32 | Serum High | | 0.00060087 | N/A | N/A | N/A | <Blank | N/A |
| 64 | Serum High | | 0.00072885 | N/A | N/A | N/A | <Blank | N/A |

This table shows the results of parallelism experiments for each assay. In the header, 'mean count' represents the raw Lumipulse reading, 'mean signal' is used for MSD – Lilly, MSD – S-PLEX, and NULISA Singleplex, while 'AEB' is used for Simoa – ALZpath and Simoa – Janssen. For Lumipulse, initial results below the LLoQ are indicated as 'GL', and for Simoa – Janssen, results below the lowest calibration point are marked as 'below blank’.

* GL= <LLoQ for Lumipulse.

**The corrected result was obtained by multiplying the dilution factor by the measured result to account for sample dilution

*Abbreviations: CV, coefficient of variation; NA, not available; AEB,* a*verage number of enzymes per bead*

**Supplementary Table 9.** The summary of the dilution linearity experiments

| **Dilution rate** | **Sample Type** | **Mean Count/Signal/AEB** | **CV%** | **Result** | **Corrected result** | **Remarks** | **Recovery%** |
| --- | --- | --- | --- | --- | --- | --- | --- |
| **Lumipulse** |  |  |  |  |  |  |  |
| 1 | Plasma Low | 23112.5 | 0.10 | 4.118 | 4.118 |  |  |
| 2 | Plasma Low | 10562.5 | 22.76 | 1.827 | 3.654 |  | 88.73 |
| 4 | Plasma Low | 5925.5 | 34.05 | 0.893 | 3.572 |  | 86.74 |
| 8 | Plasma Low | 3756 | 22.33 | 0.456 | 3.648 |  | 88.59 |
| 16 | Plasma Low | 2408.5 | 21.20 | 0.224 | 3.576 |  | 86.84 |
| 32 | Plasma Low | 1819 | 17.14 | 0.132 | 4.224 |  | 102.57 |
| 64 | Plasma Low | 1192.5 | 31.22 | 0.039 | 2.464 | GL | N/A |
| 1 | Plasma Med | 26214 | 2.03 | 4.668 | 4.668 |  |  |
| 2 | Plasma Med | 13767.5 | 3.86 | 2.438 | 4.875 |  | 104.43 |
| 4 | Plasma Med | 7474.5 | 0.99 | 1.216 | 4.862 |  | 104.16 |
| 8 | Plasma Med | 4229.5 | 3.12 | 0.544 | 4.352 |  | 93.23 |
| 16 | Plasma Med | 2689.5 | 0.00 | 0.269 | 4.304 |  | 92.20 |
| 32 | Plasma Med | 1463.5 | 35.58 | 0.078 | 2.480 |  | 53.13 |
| 64 | Plasma Med | 1294 | 9.43 | 0.053 | 3.360 |  | 71.98 |
| 1 | Plasma High | 28353 | 1.09 | 5.050 | 5.050 |  |  |
| 2 | Plasma High | 15277 | 0.23 | 2.716 | 5.431 |  | 107.54 |
| 4 | Plasma High | 8622,5 | 2.78 | 1.449 | 5.794 |  | 114.73 |
| 8 | Plasma High | 4644,5 | 0.11 | 0.627 | 5.012 |  | 99.25 |
| 16 | Plasma High | 2706 | 5.47 | 0.272 | 4.344 |  | 86.02 |
| 32 | Plasma High | 1685 | 15.85 | 0.112 | 3.568 |  | 70.65 |
| 64 | Plasma High | 1548 | 7.03 | 0.091 | 5.792 |  | 114.69 |
| 1 | Serum Low | 23369 | 0.83 | 4.188 | 4.188 |  |  |
| 2 | Serum Low | 12609 | 0.93 | 2.207 | 4.413 |  | 105.39 |
| 4 | Serum Low | 7158 | 0.25 | 1.148 | 4.592 |  | 109.66 |
| 8 | Serum Low | 3515 | 9.35 | 0.386 | 3.084 |  | 73.65 |
| 16 | Serum Low | 2275 | 10.13 | 0.189 | 3.016 |  | 72.02 |
| 32 | Serum Low | 1389 | 40.79 | 0.052 | 1.664 |  | 39.74 |
| 64 | Serum Low | 1194 | 10.35 | 0.041 | 2.624 |  | 62.66 |
| 1 | Serum Med | 25604 | 5.44 | 4.560 | 4.560 |  |  |
| 2 | Serum Med | 13828 | 3.21 | 2.449 | 4.897 |  | 107.40 |
| 4 | Serum Med | 7431 | 2.05 | 1.207 | 4.826 |  | 105.84 |
| 8 | Serum Med | 4244,5 | 2.33 | 0.547 | 4.376 |  | 95.98 |
| 16 | Serum Med | 2518,5 | 16.43 | 0.241 | 3.856 |  | 84.57 |
| 32 | Serum Med | 1464,5 | 34.45 | 0.078 | 2.496 |  | 54.74 |
| 64 | Serum Med | 1294 | 9.43 | 0.053 | 3.360 |  | 73.69 |
| 1 | Serum High | 27605 | 4.47 | 4.917 | 4.917 |  |  |
| 2 | Serum High | 14787 | 0.94 | 2.626 | 5.251 |  | 106.8 |
| 4 | Serum High | 8235 | 6.55 | 1.371 | 5.482 |  | 111.5 |
| 8 | Serum High | 4578,5 | 0.23 | 0.613 | 4.904 |  | 99.75 |
| 16 | Serum High | 2764,5 | 3.77 | 0.282 | 4.504 |  | 91.61 |
| 32 | Serum High | 1667,5 | 5.87 | 0.109 | 3.472 |  | 70.62 |
| 64 | Serum High | 1217,5 | 34.49 | 0.041 | 2.624 |  | 53.37 |
| **MSD – Lilly** |  |  |  |  |  |  |  |
| 1 | Plasma Low | 1332 | 1.83 | 4.498 | 4.498 |  |  |
| 2 | Plasma Low | 755 | 1.19 | 2.499 | 4.999 |  | 111.14 |
| 4 | Plasma Low | 423 | 3.27 | 1.322 | 5.286 |  | 117.54 |
| 8 | Plasma Low | 292 | 3.04 | 0.849 | 6.794 |  | 151.05 |
| 16 | Plasma Low | 237 | 3.23 | 0.647 | 10.355 |  | 230.22 |
| 32 | Plasma Low | 192 | 1.66 | 0.478 | 15.298 |  | 340.12 |
| 64 | Plasma Low | 186 | 9.89 | 0.456 | 29.154 |  | 648.21 |
| 1 | Plasma Med | 1340 | 2.09 | 4.523 | 4.523 |  |  |
| 2 | Plasma Med | 827 | 1.17 | 2.749 | 5.499 |  | 121.56 |
| 4 | Plasma Med | 446 | 3.07 | 1.404 | 5.616 |  | 124.16 |
| 8 | Plasma Med | 289 | 3.70 | 0.838 | 6.706 |  | 148.25 |
| 16 | Plasma Med | 221 | 8.03 | 0.588 | 9.406 |  | 207.94 |
| 32 | Plasma Med | 189 | 6.24 | 0.467 | 14.938 |  | 330.23 |
| 64 | Plasma Med | 182 | 5.42 | 0.441 | 28.196 |  | 623.34 |
| 1 | Plasma High | 1260 | 0.52 | 4.249 | 4.249 |  |  |
| 2 | Plasma High | 819 | 1.91 | 2.721 | 5.443 |  | 128.11 |
| 4 | Plasma High | 505 | 0.16 | 1.615 | 6.460 |  | 152.04 |
| 8 | Plasma High | 300 | 4.42 | 0.877 | 7.013 |  | 165.06 |
| 16 | Plasma High | 246 | 9.20 | 0.680 | 10.885 |  | 256.20 |
| 32 | Plasma High | 185 | 3.51 | 0.454 | 14.519 |  | 341.72 |
| 64 | Plasma High | 181 | 11.55 | 0.437 | 27.953 |  | 657.92 |
| 1 | Serum Low | 1443 | 8.69 | 4.755 | 4.755 |  |  |
| 2 | Serum Low | 892 | 2.43 | 2.885 | 5.770 |  | 121.34 |
| 4 | Serum Low | 735 | 8.99 | 2.347 | 9.390 |  | 197.47 |
| 8 | Serum Low | 549 | 41.26 | 1.707 | 13.656 |  | 287.20 |
| 16 | Serum Low | 514 | 1.85 | 1.588 | 25.404 |  | 534.25 |
| 32 | Serum Low | 422 | 28.60 | 1.266 | 40.508 |  | 851.92 |
| 64 | Serum Low | 363 | 37.29 | 1.061 | 67.933 |  | 1428.67 |
| 1 | Serum Med | 1416 | 4.46 | 4.664 | 4.664 |  |  |
| 2 | Serum Med | 1058 | 2.23 | 3.452 | 6.905 |  | 148.04 |
| 4 | Serum Med | 908 | 15.19 | 2.939 | 11.757 |  | 252.08 |
| 8 | Serum Med | 827 | 14.81 | 2.662 | 21.299 |  | 456.68 |
| 16 | Serum Med | 619 | 7.25 | 1.950 | 31.206 |  | 669.10 |
| 32 | Serum Med | 500 | 9.72 | 1.537 | 49.197 |  | 1054.85 |
| 64 | Serum Med | 460 | 11.77 | 1.399 | 89.509 |  | 1919.19 |
| 1 | Serum High | 1349 | 9.97 | 4.437 | 4.437 |  |  |
| 2 | Serum High | 1165 | 1.07 | 3.814 | 7.628 |  | 171.91 |
| 4 | Serum High | 799 | 2.55 | 2.567 | 10.268 |  | 231.40 |
| 8 | Serum High | 700 | 1.53 | 2.229 | 17.833 |  | 401.90 |
| 16 | Serum High | 656 | 0.70 | 2.078 | 33.245 |  | 749.25 |
| 32 | Serum High | 499 | 2.23 | 1.536 | 49.144 |  | 1107.55 |
| 64 | Serum High | 369 | 3.19 | 1.083 | 69.335 |  | 1562.58 |
| **MSD – S-PLEX** |  |  |  |  |  |  |  |
| 1 | Plasma Low | 4749 | 70.99 | 100.884 | 100.884 |  |  |
| 2 | Plasma Low | 1537 | 40.55 | 31.886 | 63.773 |  | 63.21 |
| 4 | Plasma Low | 777 | 52.35 | 15.205 | 60.820 |  | 60.29 |
| 8 | Plasma Low | 475 | 35.95 | 8.523 | 68.187 |  | 67.59 |
| 16 | Plasma Low | 272 | 32.00 | 3.954 | 63.263 |  | 62.71 |
| 32 | Plasma Low | 292 | 36.91 | 4.417 | 141.337 |  | 140.10 |
| 64 | Plasma Low | 98 | N/A | 0.387 | 24.785 |  | 24.57 |
| 1 | Plasma Med | 3991 | 1.89 | 84.900 | 84.900 |  |  |
| 2 | Plasma Med | 1527 | 10.18 | 31.680 | 63.359 |  | 74.63 |
| 4 | Plasma Med | 723 | 2.01 | 14.041 | 56.163 |  | 66.15 |
| 8 | Plasma Med | 409 | 10.58 | 7.040 | 56.322 |  | 66.34 |
| 16 | Plasma Med | 232 | 6.31 | 3.059 | 48.948 |  | 57.65 |
| 32 | Plasma Med | 164 | 3.28 | 1.492 | 47.749 |  | 56.24 |
| 64 | Plasma Med | 136 | 7.69 | 0.856 | 54.758 |  | 64.50 |
| 1 | Plasma High | 5438 | 16.0 | 115.801 | 115.800 |  |  |
| 2 | Plasma High | 1958 | 12.26 | 41.070 | 82.140 |  | 70.93 |
| 4 | Plasma High | 858 | 12.38 | 17.012 | 68.050 |  | 58.76 |
| 8 | Plasma High | 512 | 14.35 | 9.342 | 74.740 |  | 64.54 |
| 16 | Plasma High | 307 | 19.84 | 4.746 | 75.940 |  | 65.58 |
| 32 | Plasma High | 216 | 57.55 | 2.691 | 86.110 |  | 74.36 |
| 64 | Plasma High | 188 | 37.30 | 2.042 | 130.710 |  | 112.87 |
| 1 | Serum Low | 1825 | N/A | 80.512 | 80.512 |  |  |
| 2 | Serum Low | 987 | N/A | 42.432 | 84.864 |  | 105.40 |
| 4 | Serum Low | 398 | N/A | 14.969 | 59.875 |  | 74.37 |
| 8 | Serum Low | 259 | N/A | 7.902 | 63.213 |  | 78.51 |
| 16 | Serum Low | 166 | N/A | 3.009 | 48.151 |  | 59.81 |
| 32 | Serum Low | 118 | N/A | 0.559 | 17.885 |  | 22.21 |
| 64 | Serum Low | 100 | N/A | N/A | N/A |  | N/A |
| 1 | Serum Med | 4384 | 3.74 | 98.448 | 98.448 |  |  |
| 2 | Serum Med | 1630 | 2.48 | 36.417 | 72.833 |  | 73.98 |
| 4 | Serum Med | 2176 | 89.34 | 48.453 | 193.812 |  | 196.87 |
| 8 | Serum Med | 964 | 86.94 | 20.574 | 164.595 |  | 167.19 |
| 16 | Serum Med | 558 | 87.12 | 10.833 | 173.323 |  | 176.06 |
| 32 | Serum Med | 359 | 103.24 | 5.892 | 188.543 |  | 191.52 |
| 64 | Serum Med | 209 | 108.52 | 2.030 | 129.889 |  | 131.94 |
| 1 | Serum High | 4348 | 22.19 | 97.586 | 97.586 |  |  |
| 2 | Serum High | 1740 | 2.39 | 38.962 | 77.925 |  | 79.85 |
| 4 | Serum High | 911 | 7.20 | 19.509 | 78.034 |  | 79.96 |
| 8 | Serum High | 515 | 2.66 | 9.883 | 79.068 |  | 81.02 |
| 16 | Serum High | 278 | 14.68 | 3.886 | 62.171 |  | 63.71 |
| 32 | Serum High | 200 | 14.60 | 1.835 | 58.709 |  | 60.16 |
| 64 | Serum High | 141 | N/A | 0.443 | 28.354 |  | 29.06 |
| NULISA Singleplex |  |  |  |  |  |  |  |
| 1 | Plasma Low | 4502.53 | 0.70 | 14.027 | 14.027 |  |  |
| 2 | Plasma Low | 894.33 | 2.10 | 2.931 | 5.861 |  | 41.78 |
| 4 | Plasma Low | 315.75 | 1.88 | 1.056 | 4.222 |  | 30.10 |
| 8 | Plasma Low | 154.98 | 3.49 | 0.516 | 4.130 |  | 29.44 |
| 16 | Plasma Low | 78.70 | 16.33 | 0.253 | 4.052 |  | 28.89 |
| 32 | Plasma Low | 22.31 | 40.79 | 0.052 | 1.664 |  | 11.86 |
| 64 | Plasma Low | 11.31 | 49.50 | 0.010 | 0.640 |  | 4.56 |
| 1 | Plasma Med | 3424.15 | 0.90 | 10.767 | 10.767 |  |  |
| 2 | Plasma Med | 975.71 | 2.86 | 3.190 | 6.380 |  | 59.26 |
| 4 | Plasma Med | 367.32 | 3.23 | 1.226 | 4.904 |  | 45.55 |
| 8 | Plasma Med | 169.74 | 5.87 | 0.567 | 4.532 |  | 42.09 |
| 16 | Plasma Med | 91.80 | 3.31 | 0.299 | 4.784 |  | 44.43 |
| 32 | Plasma Med | 19.76 | 1.66 | 0.043 | 1.360 |  | 12.63 |
| 64 | Plasma Med | 9.95 | N/A | 0.011 | 0.672 |  | 6.24 |
| 1 | Plasma High | 3563.88 | 2.46 | 11.191 | 11.191 |  |  |
| 2 | Plasma High | 1155.88 | 11.73 | 3.761 | 7.521 |  | 67.21 |
| 4 | Plasma High | 375.65 | 5.81 | 1.254 | 5.014 |  | 44.81 |
| 8 | Plasma High | 248.50 | 6.80 | 0.728 | 5.820 |  | 52.01 |
| 16 | Plasma High | 90.24 | 10.15 | 0.254 | 4.068 |  | 36.35 |
| 32 | Plasma High | 21.25 | 11.79 | 0.042 | 1.344 |  | 12.01 |
| 64 | Plasma High | 13.53 | 92.23 | 0.017 | 1.104 |  | 9.87 |
| 1 | Serum Low | 3611.60 | 10.84 | 10.268 | 10.268 |  |  |
| 2 | Serum Low | 902.03 | 12.20 | 2.628 | 5.256 |  | 51.18 |
| 4 | Serum Low | 326.67 | 4.43 | 0.958 | 3.832 |  | 37.32 |
| 8 | Serum Low | 171.90 | 3.54 | 0.500 | 4.000 |  | 38.96 |
| 16 | Serum Low | 92.63 | 17.11 | 0.298 | 4.760 |  | 46.36 |
| 32 | Serum Low | 17.48 | 11.79 | 0.030 | 0.960 |  | 9.35 |
| 64 | Serum Low | 13.21 | 4.16 | 0.017 | 1.088 |  | 10.60 |
| 1 | Serum Med | 3685.79 | 12.36 | 10.475 | 10.475 |  |  |
| 2 | Serum Med | 954.41 | 6.27 | 2.778 | 5.557 |  | 53.05 |
| 4 | Serum Med | 350.91 | 3.47 | 1.029 | 4.117 |  | 39.30 |
| 8 | Serum Med | 173.90 | 10.98 | 0.506 | 4.046 |  | 38.63 |
| 16 | Serum Med | 89.21 | 12.24 | 0.251 | 4.020 |  | 38.38 |
| 32 | Serum Med | 43.41 | 6.04 | 0.111 | 3.560 |  | 33.99 |
| 64 | Serum Med | 28.68 | 8.13 | 0.065 | 4.176 |  | 39.87 |
| 1 | Serum High | 3595.57 | 0.38 | 10.224 | 10.224 |  |  |
| 2 | Serum High | 1088.81 | 0.92 | 3.164 | 6.327 |  | 61.88 |
| 4 | Serum High | 430.84 | 3.75 | 1.264 | 5.054 |  | 49.43 |
| 8 | Serum High | 174.85 | 0.07 | 0.509 | 4.070 |  | 39.81 |
| 16 | Serum High | 87.29 | 2.45 | 0.245 | 3.924 |  | 38.38 |
| 32 | Serum High | 28.70 | 36.30 | 0.065 | 2.088 |  | 20.42 |
| 64 | Serum High | 10.69 | 38.57 | 0.008 | 0.528 |  | 5.16 |
| **Simoa – ALZpath** | |  |  |  |  |  |  |
| 1 | Plasma Low | 4.42500066 | 3.09 | 13.087 | 13.087 |  |  |
| 2 | Plasma Low | 2.45858213 | 10.11 | 7.374 | 14.748 |  | 112.69 |
| 4 | Plasma Low | 1.12948626 | 6.25 | 3.455 | 13.819 |  | 105.59 |
| 8 | Plasma Low | 0.59452135 | 9.30 | 1.846 | 14.767 |  | 112.84 |
| 16 | Plasma Low | 0.31263835 | 12.77 | 0.982 | 15.715 |  | 120.08 |
| 32 | Plasma Low | 0.14034855 | 6.44 | 0.443 | 14.173 |  | 108.30 |
| 64 | Plasma Low | 0.07871039 | 1.41 | 0.246 | 15.716 |  | 120.09 |
| 1 | Plasma Med | 4.63388979 | 2.06 | 13.691 | 13.691 |  |  |
| 2 | Plasma Med | 2.44280153 | 6.35 | 7.328 | 14.656 |  | 107.05 |
| 4 | Plasma Med | 1.12446048 | 3.17 | 3.440 | 13.759 |  | 100.50 |
| 8 | Plasma Med | 0.5831879 | 8.76 | 1.811 | 14.492 |  | 105.85 |
| 16 | Plasma Med | 0.24865244 | 11.32 | 0.783 | 12.534 |  | 91.55 |
| 32 | Plasma Med | 0.14626046 | 3.49 | 0.462 | 14.774 |  | 107.91 |
| 64 | Plasma Med | 0.07314976 | 0.33 | 0.228 | 14.563 |  | 106.37 |
| 1 | Plasma High | 4.23768962 | 2.75 | 12.546 | 12.546 |  |  |
| 2 | Plasma High | 2.15875768 | 2.81 | 6.496 | 12.993 |  | 103.56 |
| 4 | Plasma High | 1.09322833 | 7.96 | 3.346 | 13.386 |  | 106.70 |
| 8 | Plasma High | 0.52240863 | 1.22 | 1.627 | 13.012 |  | 103.72 |
| 16 | Plasma High | 0.29563193 | 4.77 | 0.930 | 14.873 |  | 118.55 |
| 32 | Plasma High | 0.13328538 | 7.50 | 0.420 | 13.455 |  | 107.25 |
| 64 | Plasma High | 0.06732068 | 5.33 | 0.209 | 13.351 |  | 106.42 |
| 1 | Serum Low | 3.90292151 | 5.06 | 11.576 | 11.576 |  |  |
| 2 | Serum Low | 2.08412724 | 3.42 | 6.277 | 12.555 |  | 108.45 |
| 4 | Serum Low | 1.11420426 | 2.66 | 3.409 | 13.637 |  | 117.80 |
| 8 | Serum Low | 0.50348336 | 3.25 | 1.569 | 12.55 |  | 108.41 |
| 16 | Serum Low | 0.26935259 | 4.34 | 0.848 | 13.567 |  | 117.19 |
| 32 | Serum Low | 0.12753067 | 3.34 | 0.402 | 12.869 |  | 111.17 |
| 64 | Serum Low | 0.07018188 | 4.02 | 0.218 | 13.947 |  | 120.48 |
| 1 | Serum Med | 3.85492643 | 21.81 | 11.435 | 11.435 |  |  |
| 2 | Serum Med | 2.09363829 | 5.61 | 6.305 | 12.610 |  | 110.28 |
| 4 | Serum Med | 1.07865402 | 4.69 | 3.303 | 13.212 |  | 115.54 |
| 8 | Serum Med | 0.57326979 | 13.77 | 1.781 | 14.249 |  | 124.61 |
| 16 | Serum Med | 0.28105884 | 2.28 | 0.884 | 14.149 |  | 123.74 |
| 32 | Serum Med | 0.13743434 | 4.19 | 0.434 | 13.877 |  | 121.36 |
| 64 | Serum Med | 0.0798494 | 4.73 | 0.249 | 15.951 |  | 139.50 |
| 1 | Serum High | 3.53301387 | 7.74 | 10.504 | 10.504 |  |  |
| 2 | Serum High | 2.35630457 | 2.64 | 7.075 | 14.150 |  | 134.72 |
| 4 | Serum High | 1.00561763 | 0.92 | 3.085 | 12.339 |  | 117.47 |
| 8 | Serum High | 0.49726098 | 11.79 | 1.550 | 12.397 |  | 118.03 |
| 16 | Serum High | 0.26781062 | 4.14 | 0.843 | 13.49 |  | 128.43 |
| 32 | Serum High | 0.13174873 | 1.23 | 0.416 | 13.299 |  | 126.61 |
| 64 | Serum High | 0.01117252 | 27.17 | 0.022 | 1.389 |  | 13.23 |
| **Simoa – Janssen** | |  |  |  |  |  |  |
| 1 | Plasma Low | 0.04461857 | 7.90 | 3.372 | 3.372 |  |  |
| 2 | Plasma Low | 0.02518205 | 1.09 | 1.906 | 3.812 |  | 113.04 |
| 4 | Plasma Low | 0.01251143 | 16.63 | 0.928 | 3.713 |  | 110.11 |
| 8 | Plasma Low | 0.00736629 | 6.97 | 0522 | 4.173 |  | 123.77 |
| 16 | Plasma Low | 0.00567109 | N/A | 0.385 | 6.165 |  | 182.84 |
| 32 | Plasma Low | 0.00548901 | 74.40 | 0.369 | 11.793 |  | 349.75 |
| 64 | Plasma Low | 0.00207555 | N/A | 0.088 | 5.636 |  | 167.13 |
| 1 | Plasma Med | 0.0429325 | 19.70 | 3.245 | 3.245 |  |  |
| 2 | Plasma Med | 0.02165574 | 16.35 | 1.636 | 3.272 |  | 100.82 |
| 4 | Plasma Med | 0.01320596 | 12.98 | 0.983 | 3.930 |  | 121.12 |
| 8 | Plasma Med | 0.00668722 | 54.80 | 0.466 | 3.727 |  | 114.86 |
| 16 | Plasma Med | 0.00521846 | 51.28 | 0.348 | 5.564 |  | 171.46 |
| 32 | Plasma Med | 0.00296768 | 71.00 | 0.163 | 5.204 |  | 160.39 |
| 64 | Plasma Med | 0.00202173 | 82.19 | 0.083 | 5.304 | <Blank |  |
| 1 | Plasma High | 0.04235372 | 9.94 | 3.202 | 3.202 |  |  |
| 2 | Plasma High | 0.02482625 | 25.75 | 1.878 | 3.755 |  | 117.26 |
| 4 | Plasma High | 0.01404339 | 18.20 | 1.048 | 4.191 |  | 130.88 |
| 8 | Plasma High | 0.00858865 | 36.63 | 0.618 | 4.947 |  | 154.48 |
| 16 | Plasma High | 0.00399867 | N/A | 0.249 | 3.983 |  | 124.38 |
| 32 | Plasma High | 0.00272702 | 54.51 | 0.143 | 4.572 |  | 142.76 |
| 64 | Plasma High | 0.00326941 | 96.80 | 0.187 | 11.954 | <Blank |  |
| 1 | Serum Low | 0.03842393 | 18.42 | 2.907 | 2.907 |  |  |
| 2 | Serum Low | 0.0211009 | 0.30 | 1.594 | 3.187 |  | 109.64 |
| 4 | Serum Low | 0.01250202 | 22.67 | 0.927 | 3.709 |  | 127.59 |
| 8 | Serum Low | 0.00639129 | 50.68 | 0442 | 3.539 |  | 121.74 |
| 16 | Serum Low | 0.0040423 | 24.27 | 0.252 | 4.038 |  | 138.91 |
| 32 | Serum Low | 0.0034029 | 12.89 | 0.200 | 6.390 |  | 219.81 |
| 64 | Serum Low | 0.00221618 | 46.77 | 0.100 | 6.391 |  | 219.84 |
| 1 | Serum Med | 0.03285883 | 8.70 | 2.488 | 2.488 |  |  |
| 2 | Serum Med | 0.0181564 | 15.25 | 1.367 | 2.733 |  | 109.85 |
| 4 | Serum Med | 0.01327914 | 33.36 | 0.987 | 3.95 |  | 158.73 |
| 8 | Serum Med | 0.00897974 | 20.04 | 0.650 | 5.200 |  | 208.97 |
| 16 | Serum Med | 0.00424814 | 18.88 | 0.269 | 4.309 |  | 173.19 |
| 32 | Serum Med | 0.00303204 | 0.33 | 0.169 | 5.403 |  | 217.13 |
| 64 | Serum Med | 0.00194206 | 25.85 | 0.077 | 4.899 |  | 196.87 |
| 1 | Serum High | 0.03299195 | 6.41 | 2.498 | 2.498 |  |  |
| 2 | Serum High | 0.0195457 | 9.33 | 1.474 | 2.948 |  | 84.75 |
| 4 | Serum High | 0.01132691 | 34.15 | 0.835 | 3.338 |  | 133.62 |
| 8 | Serum High | 0.00789018 | 5.10 | 0.564 | 4.508 |  | 180.44 |
| 16 | Serum High | 0.00419288 | N/A | 0.265 | 4238 |  | 169.64 |
| 32 | Serum High | 0.00236635 | 22.94 | 0.113 | 3.609 |  | 144.45 |
| 64 | Serum High | 0.00253096 | 49.34 | 0.126 | 8.094 |  | 323.96 |

This table shows the results of dilution linearity experiments for each assay. In the header, 'mean count' represents the raw Lumipulse reading, 'mean signal' is used for MSD – Lilly, MSD – S-PLEX, and NULISA Singleplex , while 'AEB' is used for Simoa – ALZpath and Simoa – Janssen. For Lumipulse, initial results below the LLoQ are indicated as 'GL', and for Simoa – Janssen, results below the lowest calibration point are marked as 'below blank’.

* GL= <LLoQ for Lumipulse.

**The corrected result was obtained by multiplying the dilution factor by the measured result to account for sample dilution

*Abbreviations: CV, coefficient of variation;; NA, not available; AEB,* a*verage number of enzymes per bead*

**Supplementary Table 10.** Spike recovery

|  |  |  | **Plasma** |  |  |  | **Serum** |  |
| --- | --- | --- | --- | --- | --- | --- | --- | --- |
| **Assay** | **Sample** | **Measured c.** | **Expected c.** | **% Recovery** |  | **Measured c.** | **Expected c.** | **% Recovery** |
|  | Low | 0.1 | 0.972 | 124.14 |  | 0.095 | 0.074 | 128.38 |
| **Lumipulse** | Medium | 0.871 | 0.783 | 111.24 |  | 0.454 | 0.435 | 104.37 |
|  | High | 1.818 | 1.638 | 110.99 |  | 1.394 | 1.22 | 114.26 |
|  | Low | 0.360 | 0.333 | 108.09 |  | 1.220 | 1.064 | 114.62 |
| **MSD - Lilly** | Medium | 0.804 | 0.774 | 103.87 |  | 1.786 | 1.338 | 133.47 |
|  | High | 1.234 | 1.469 | 84.00 |  | 1.810 | 1.385 | 130.68 |
|  | Low | 5.18 | 6.48 | 79.85 |  | 1.92 | 1.79 | 107.56 |
| **MSD - S-PLEX** | Medium | 22.80 | 28.76 | 79.28 |  | 6.98 | 5.91 | 117.95 |
|  | High | 41.93 | 55.75 | 75.20 |  | 18.48 | 25.31 | 72.99 |
|  | Low | 0.378 | 1.957 | 19.32 |  | 0.283 | 1.446 | 19.56 |
| NULISA Singleplex | Medium | 1.235 | 2.474 | 49.93 |  | 0.489 | 1.106 | 44.16 |
|  | High | 2.469 | 4.471 | 55.21 |  | 1.189 | 2.159 | 55.06 |
|  | Low | 0.35 | 0.33 | 106.06 |  | 0.12 | 0.14 | 86.51 |
| **Simoa - ALZpath** | Medium | 1.34 | 1.29 | 103.88 |  | 0.29 | 0.39 | 75.03 |
|  | High | 2.74 | 2.62 | 104.58 |  | 0.62 | 0.98 | 63.92 |
|  | Low | 0.050 | 0.040 | 124.40 |  | 0.020 | 0.017 | 115.34 |
| **Simoa - Janssen** | Medium | 0.166 | 0.129 | 128.95 |  | 0.061 | 0.051 | 119.65 |
|  | High | 0.388 | 0.397 | 97.69 |  | 0.175 | 0.156 | 112.25 |

**Supplementary** **Table 11**. Sample stability experiment results

|  |  | **Cycle** | | | | | |
| --- | --- | --- | --- | --- | --- | --- | --- |
| **Assay** | **Sample** | **#1** | **#2** | **#3** | **#4** | **#5** | **#6** |
| Lumipulse | Plasma low | 0.124 (-2.89) | 0.132 (2.98) | 0.130 (1.48) | 0.136 (5.84) | 0.131 (2.23) | 0.115 (-11.43) |
|  | Plasma medium | 0.205 (4.11) | 0.202 (2.44) | 0.204 (3.64) | 0.191 (-2.92) | 0.189 (-4.01) | 0.189 (-4.01) |
|  | Plasma high | 0.738 (9.68) | 0.687 (2.97) | 0.701 (4.98) | 0.671 (0.73) | 0.630 (-5.73) | 0.571 (-16.75) |
|  | Serum low | 0.070 (9.23) | 0.068 (7.23) | 0.075 (15.89) | 0.060 (-6.02) | 0.056 (-12.65) | 0.051 (-24.92) |
|  | Serum medium | 0.249 (11.64) | 0.218 (-0.73) | 0.225 (2.41) | 0.214 (-2.61) | 0.209 (-5.32) | 0.204 (-7.90) |
|  | Serum high | 0.906 (8.52) | 0.841 (1.39) | 0.862 (3.79) | 0.829 (0.02) | 0.763 (-8.63) | 0.773 (-7.22) |
| MSD - Lilly | Plasma low | 0.289 (-1.60) | 0.303 (3.40) | 0.289 (-1.61) | 0.278 (-5.51) | 0.295 (0.61) | 0.306 (4.08) |
|  | Plasma medium | 0.421 (-3.36) | 0.442 (1.59) | 0.435 (0.17) | 0.442 (1.60) | 0.425 (-2.33) | 0.444 (2.07) |
|  | Plasma high | 0.734 (-9.64) | 0.808 (0.30) | 0.814 (1.07) | 0.822 (2.08) | 0.827 (2.57) | 0.827 (2.57) |
|  | Serum low | 0.663 (-38.77) | 0.613 (-50.02) | 0.791 (-16.26) | 1.111 (17.28) | 0.944 (2.64) | 1.394 (34.06) |
|  | Serum medium | 0.853 (-11.52) | 0.970 (1.91) | 1.024 (7.04) | 0.815 (-16.71) | 0.993 (4.17) | 1.054 (9.74) |
|  | Serum high | 3.018 (16.15) | 2.634 (3.93) | 2.489 (-1.67) | 2.399 (-5.52) | 2.447 (-3.43) | 2.198 (-15.16) |
| MSD –  S-PLEX | Plasma low | 3.93 (11.37) | 3.43 (-1.52) | 3.52 (1.14) | 3.31 (-5.17) | 3.62 (3.66) | 3.09 (-12.83) |
|  | Plasma medium | 7.00 (-9.19) | 8.72 (12.29) | 7.85 (2.65) | 7.50 (-2.00) | 7.66 (0.14) | 7.15 (-6.94) |
|  | Plasma high | 16.36 (-3.61) | 15.32 (-10.69) | 17.62 (3.77) | 18.70 (9.35) | 16.07 (-5.50) | 17.65 (3.96) |
|  | Serum low | 0.85 (-6.01) | 0.92 (2.19) | 1.10 (18.11) | 0.55 (-64.06) | 0.90 (-0.11) | 1.08 (16.98) |
|  | Serum medium | 3.39 (3.30) | 3.03 (-8.33) | 3.41 (3.68) | 3.42 (4.19) | 2.78 (-17.93) | 3.65 (10.14) |
|  | Serum high | 10.83 (-2.18) | 12.44 (11.08) | 11.80 (6.24) | 10.63 (-4.12) | 10.92 (-1.35) | 9.77 (-13.24) |
| Simoa - ALZpath | Plasma low | 0.228 (-5.59) | 0.252 (4.46) | 0.243 (1.04) | 0.255 (5.41) | 0.228 (-5.73) | 0.239 (-0.73) |
|  | Plasma medium | 0.541 (-1.36) | 0.562 (2.28) | 0.554 (0.86) | 0.564 (2.70) | 0.535 (-2.58) | 0.537 (-2.15) |
|  | Plasma high | 1.418 (-2.10) | 1.401 (-3.33) | 1.498 (3.38) | 1.453 (0.37) | 1.420 (-1.93) | 1.495 (3.19) |
|  | Serum low | 0.112 (2.38) | 0.112 (2.35) | 0.102 (-7.14) | 0.104 (-4.34) | 0.110 (0.61) | 0.115 (5.10) |
|  | Serum medium | 0.386 (-4.21) | 0.376 (-6.77) | 0.380 (-5.81) | 0.407 (1.23) | 0.438 (8.20) | 0.425 (5.39) |
|  | Serum high | 2.475 (-3.56) | 2.390 (-7.25) | 2.530 (-1.29) | 2.563 (0.01) | 2.753 (6.89) | 2.667 (3.90) |
| Simoa - Janssen | Plasma low | 0.027 (9.45) | 0.025 (2.15) | 0.024 (-0.37) | 0.027 (9.76) | 0.020 (-20.32) | 0.023 (-6.59) |
|  | Plasma medium | 0.046 (-4.16) | 0.046 (-4.63) | 0.048 (0.12) | 0.050 (2.59) | 0.049 (1.68) | 0.050 (3.78) |
|  | Plasma high | 0.161 (-1.50) | 0.158 (-3.19) | 0.163 (0.14) | 0.162 (-0.87) | 0.171 (4.55) | 0.164 (0.53) |
|  | Serum low | 0.007 (2.62) | 0.008 (19.13) | 0.008 (12.15) | 0.005 (-26.01) | 0.007 (-3.14) | 0.006 (-19.77) |
|  | Serum medium | 0.043 (-1.57) | 0.048 (7.28) | 0.043 (-3.49) | 0.046 (4.61) | 0.043 (-3.69) | 0.042 (-4.39) |
|  | Serum high | 0.128 (2.35) | 0.127 (1.10) | 0.121 (-3.10) | 0.134 (6.33) | 0.121 (-3.73) | 0.121 (-3.82) |

This table summarizes the results of the sample stability experiment for p-tau217 concentrations (pg/mL) measured using 5 immunoassay platforms: Lumipulse, MSD – Lilly, MSD – S-PLEX, Simoa - ALZpath, and Simoa - Janssen. The data represent the stability of plasma and serum samples across multiple freeze-thaw cycles (#1 to #6). Each freeze-thaw cycle is denoted by a column, with corresponding p-tau217 concentrations shown in the rows for low, medium, and high concentration pools of plasma and serum. For each measurement, the percentage difference from the average concentration is shown in parentheses.

**Supplementary Table 12.** Associations between quantifications on the different tube types, within assays.

| **Assay** | **Tube type** | **Passing-Bablok regression** | **Intercept CI**  **(95%)** | **Slope CI**  **(95%)** | **Spearman rho**  **(95% CI)** |
| --- | --- | --- | --- | --- | --- |
|  | Citrate | C = -0.001+0.86*E | -0.006 – 0.002 | 0.84 – 0.89^★^ | 0.99 (0.99 – 1.00) |
| Lumipulse | Li-Hep | L = 0.001+1.02*E | -0.006 – 0.012 | 0.96 – 1.08 | 0.99 (0.91 – 1.00) |
|  | Serum | S = -0.006+1.17*E | -0.011 – 0.006 | 1.05 – 1.21^★^ | 0.99 (0.96 – 1.00) |
|  | Citrate | C = -0.14+0.94*E | -0.04 – 0.02 | 0.81 – 1.04 | 0.93 (0.75 – 0.99) |
| MSD - Lilly | Li-Hep | L = -0.05+1.04*E | -0.16 – 0.04 | 0.79 – 1.39 | 0.92 (0.74 – 0.98) |
|  | Serum | S = -0.02+2.61*E | -1.36 – 0.47 | 1.06 – 6.96^★^ | 0.51 (-0.02 – 0.86) |
|  | Citrate | C = 0.28+0.78*E | -0.66 – 0.95 | 0.72 – 0.88^★^ | 0.99 (0.93 – 1.00) |
| MSD – S-PLEX | Li-Hep | L = 0.52+0.86*E | -1.41 – 0.96 | 0.81 – 1.09 | 0.98 (0.91 – 1.00) |
|  | Serum | S = -0.14+0.56*E | -1.44 – 1.66 | 0.32 – 0.72^★^ | 0.95 (0.81 – 0.99) |
|  | Citrate | C = 0.01+0.84*E | -0.05 – 0.06 | 0.77 – 0.96^★^ | 0.94 (0.75 – 0.99) |
| NULISA Singleplex | Li-Hep | L = -0.006+0.92*E | -0.11 – 0.03 | 0.86 – 1.05 | 0.96 (0.82 – 1.00) |
|  | Serum | S = 0.05+0.55*E | -0.11 – 0.18 | 0.35 – 0.71^★^ | 0.87 (0.61 – 0.96) |
|  | Citrate | C = -0.007+0.88*E | -0.03 – 0.02 | 0.79 – 0.96^★^ | 0.97 (0.88 – 0.99) |
| Simoa - ALZpath | Li-Hep | L = -0.02+1.18*E | -0.12 – 0.03 | 1.05 – 1.45^★^ | 0.87 (0.56 – 0.99) |
|  | Serum | S = -0.005+0.64*E | -0.08 – 0.04 | 0.40 – 0.83^★^ | 0.96 (0.85 – 0.99) |
|  | Citrate | C = -0.003+0.77*E | -0.003 – 0.003 | 0.71 – 0.87^★^ | 0.94 (0.72 – 0.99) |
| Simoa - Janssen | Li-Hep | L = -0.001+1.18*E | -0.01 – 0.01 | 1.00 – 1.36 | 0.96 (0.83 – 0.99) |
|  | Serum | S = 0.003+0.33*E | -0.007 – 0.01 | 0.22 – 0.50^★^ | 0.85 (0.58 – 0.94) |

The regression includes plasma EDTA quantifications as the reference method and the other tube types as the test methods. Confidence intervals (CI) at 95% were estimated using 1000 bootstrap resamples.

The * indicates multiplication.

The ^★^ indicates intercepts that are significantly different from 0 or slopes that are significantly different from 1.

*Abbreviations: C, plasma Citrate; CI, confidence interval; E, plasma EDTA; L, plasma Lithium-Heparin; S, serum.*

**Supplementary Table 13.** Blood pTau217 concentration and CV across tube types.

| **Assay** | **Tube** | **Median concentration** | ***P* value** | **Median CV** | ***P* value** |
| --- | --- | --- | --- | --- | --- |
|  | Citrate | 0.10 [0.03, 0.65] |  | 3.20 [0.28, 27.3] |  |
| Lumipulse | EDTA | 0.12 [0.03, 0.76] | 0.83 | 4.98 [0.32, 16.7] | 0.46 |
|  | Lit-Hep | 0.109 [0.03, 0.807] |  | 5.58 [0.24, 24.6] |  |
|  | Serum | 0.13 [0.03, 0.91] |  | 5.58 [0.91, 15.5] |  |
|  | Citrate | 0.2 [0.113, 1.05]* |  | 2.36 [0, 15.3] |  |
| MSD - Lilly | EDTA | 0.32 [0.14, 1.14]* | <0.0001 | 3.92 [0, 16.0] | 0.37 |
|  | Lit-Hep | 0.27 [0.17, 1.10]* |  | 3.99 [0, 12.9] |  |
|  | Serum | 0.80 [0.39, 1.42] |  | 5.74 [0.47, 28.0] |  |
|  | Citrate | 7.09 [1.25, 40.4] |  | \| 6.04 [1.44, 26.7] \| \| --- \| |  |
| MSD – S-PLEX^★^ | EDTA | 7.91 [1.57, 54.3] | 0.15 | 3.61 [0.07, 26.5] | 0.36 |
|  | Lit-Hep | 7.08 [1.91, 45.9] |  | 5.39 [0.15, 43.5] |  |
|  | Serum | 4.61 [0.42, 17.7] |  | 6.16 [0.75, 62.9] |  |
|  | Citrate | 0.64 [0.24, 4.61] |  | 6.30 [0.50, 23.1] |  |
| NULISA Singleplex | EDTA | 0.74 [0.29, 5.83] | 0.11 | 5.70 [1.20, 16.3] | 0.47 |
|  | Lit-Hep | 0.68 [0.29, 5.23] |  | 3.40 [0.50, 20.6] |  |
|  | Serum | 0.47 [0.19, 2.13] |  | 4.80 [1.00, 25.8] |  |
|  | Citrate | 0.37 [0.15, 1.72] |  | 6.21 [0.20, 43.5] |  |
| Simoa - ALZpath | EDTA | 0.46 [0.16, 1.90] | 0.03 | 4.06 [0.05, 7.51] | 0.22 |
|  | Lit-Hep | 0.67 [0.20, 1.98]* |  | 2.99 [0.43, 13.1] |  |
|  | Serum | 0.20 [0.10, 0.82] |  | \| 4.06 [0.01, 14.5] \| \| --- \| |  |
|  | Citrate | 0.04 [0.01, 0.20] |  | 5.20 [0.60, 18.6] |  |
| Simoa - Janssen | EDTA | 0.0 [0.02, 0.26]* | 0.001 | 7.00 [1.20, 30.1] | 0.13 |
|  | Lit-Hep | 0.07 [0.02, 0.35]* |  | 2.80 [0.20, 28.6] |  |
|  | Serum | 0.02 [0.01, 0.07] |  | 10.8 [0.10, 44.6] |  |

Unless otherwise specified, median (min-max) values are given. If the sample concentration was below the LLOQ, the CV was not computed in this calculation.

^★^Due to technical issues, duplicate measurements could not be performed for 7 samples (all matrices) on the MSD – S-PLEX assay.

*Median values are significantly different from serum after Dunn’s test post-hoc analysis.

*Abbreviations: CV, coefficient of variation, Lit-Hep, Lithium-Heparin.*

**Supplementary Table 14.** Assays LLOQ values on study *Phase III*.

| **Assay** | **Tube** | **LLOQ** | **Number of samples**  **below LLOQ** |
| --- | --- | --- | --- |
|  | Citrate |  | 3 |
| Lumipulse | EDTA | 0.03 | 1 |
|  | Lit-Hep |  | 1 |
|  | Serum |  | 3 |
|  | Citrate |  | 2 |
| MSD - Lilly | EDTA | 0.18 | 1 |
|  | Lit-Hep |  | 1 |
|  | Serum |  | 0 |
|  | Citrate |  | 0 |
| MSD – S-PLEX* | EDTA | 0.006 | 0 |
|  | Lit-Hep |  | 0 |
|  | Serum |  | 0 |
|  | Citrate |  | 1 |
| NULISA Singleplex | EDTA | 0.295 | 0 |
|  | Lit-Hep |  | 1 |
|  | Serum |  | 4 |
|  | Citrate |  | 0 |
| Simoa - ALZpath | EDTA | 0.007 | 0 |
|  | Lit-Hep |  | 0 |
|  | Serum |  | 0 |
|  | Citrate |  | 0 |
| Simoa - Janssen | EDTA | 0.0038 | 0 |
|  | Lit-Hep |  | 0 |
|  | Serum |  | 0 |

^*^Due to technical issues, duplicate measurements could not be performed for 7 samples (all matrices) on the MSD – S-PLEX assay.

*Abbreviations: Lit-Hep, Lithium-Heparin; LLOQ, lower limit of quantification.*

**Supplementary Figure 1. Overview of the study design and phases**

**Supplementary Figure 2.** Relationship between CV and sample concentration on *Phase II* LLoQ samples.

**References**

1. Feng W, Beer JC, Hao Q, Ariyapala IS, Sahajan A, Komarov A, et al. Nulisa: A proteomic liquid biopsy platform with attomolar sensitivity and high multiplexing. Nature Communications 2023;14:1:7238.

2. Ashton NJ, Keshavan A, Brum WS, Andreasson U, Arslan B, Droescher M, et al. The alzheimer's association global biomarker standardization consortium (gbsc) plasma phospho‐tau round robin study. Alzheimer's & Dementia 2025;21:2:e14508.

3. Andreasson U, Perret-Liaudet A, van Waalwijk van Doorn LJ, Blennow K, Chiasserini D, Engelborghs S, et al. A practical guide to immunoassay method validation. Frontiers in neurology 2015;6:179.

4. 15189:2022. I. Medical laboratories – requirements for quality and competence (2022).

5. ISO5725-2. Accuracy(truenessandprecision)ofmeasurementmethodsand

results–part2:Basicmethodforthedeterminationofrepeatabilityand

reproducibilityofastandardmeasurementmethod (1994).

6. Ashton NJ, Brum WS, Di Molfetta G, Benedet AL, Arslan B, Jonaitis E, et al. Diagnostic accuracy of a plasma phosphorylated tau 217 immunoassay for alzheimer disease pathology. JAMA neurology 2024;81:3:255-63.
